# Integrated Salmon Hatcheries Can Pose Less Genetic Risk to Wild Populations Than Segregated Programs, Given Imperfect Implementation

**DOI:** 10.1111/eva.70184

**Published:** 2025-12-02

**Authors:** Jack H. Buckner, Michael J. Ford, Marissa L. Baskett

**Affiliations:** ^1^ Department of Environmental Science and Policy University of California Davis Davis California USA; ^2^ College of Earth oceans and Atmospheric Science Oregon State University Corvallis Oregon USA; ^3^ Conservation Biology Division, Northwest Fisheries Science Center, National Marine Fisheries Service National Oceanic and Atmospheric Administration Seattle Washington USA

**Keywords:** artificial propagation, gene flow, local adaptation, Pacific Salmon (Oncorhynchus spp.), population genetics—theoretical, quantitative genetics

## Abstract

Hatchery programs can provide fishery and conservation benefits, but can also inadvertently threaten wild populations through genetic and ecological interactions. Two common, and non‐mutually exclusive, strategies for mitigating the genetic impacts of hatchery programs on wild populations are reducing the number of hatchery‐origin fish spawning in the wild and integrating wild‐origin individuals into the hatchery broodstock. We compared the robustness of these two strategies to imperfect implementation (variation around target proportions of hatchery‐origin spawners in the wild and wild‐origin brood stock) using a quantitative population genetic model. Simulations revealed that incorporating wild‐origin broodstock was more robust to both short‐ and long‐term implementation errors compared to minimizing hatchery‐origin spawners in the wild. Furthermore, relatively low levels of hatchery integration were required to achieve most of the increase in robustness, provided that the average proportion of hatchery‐origin spawners was correspondingly low. We checked these findings against empirically observed levels of implementation error by parametrizing the model using data from hatchery programs in Washington and Oregon. These findings suggest that integrated hatchery programs can pose a smaller genetic risk to wild populations than segregated programs, given realistic levels of implementation error.

## 1 Introduction

Many salmon fisheries throughout the world are supported by large‐scale releases of hatchery‐raised fish (Ruggerone and Irvine 2018; Nelson et al. 2019; McMillan et al. 2023). These hatchery programs can support economically and culturally important fisheries and can also serve as conservation tools to preserve threatened and endangered populations (Mobrand et al. 2005; Osborne et al. 2020). However, hatchery programs also have unintended genetic and ecological consequences that threaten naturally reproducing populations (Naish et al. 2007; Laikre et al. 2010). Over several generations, hatchery‐raised populations can adapt to captivity through the process of domestication. Salmon released from hatcheries typically have lower reproductive success when they return to spawn in the wild (Araki et al. 2007; Christie et al. 2014; Koch and Narum 2021), partially caused by fitness tradeoffs between captive and natural environments (Christie et al. 2012). Because of these fitness tradeoffs, gene flow from hatchery populations to naturally reproducing ones can reduce the overall fitness and threaten the viability of wild salmonid populations (Tufto 2001; Baskett and Waples 2013; Willoughby and Christie 2019).

The impacts of hatchery production on wild population fitness caused by domestication selection can be mitigated by two primary strategies: reducing gene flow from the hatchery to the wild and introducing naturally occurring fish into the hatchery broodstock (Ford 2002). Reducing gene flow from the hatchery into the wild can limit the extent to which domestication in the hatchery influences wild population fitness (Tufto 2001; Baskett and Waples 2013; Buckner et al. 2024). Increasing gene flow from the wild population to the hatchery can reduce domestication of the hatchery population, which in turn mitigates the fitness effect of gene flow from the hatchery should it occur (Ford 2002; Janowitz‐Koch et al. 2019; Larsen et al. 2019). Programs that rely on limiting gene flow into wild populations and do not integrate wild‐origin fish into the hatchery broodstock are called segregated (or isolated) programs, and hatcheries that use wild‐origin broodstock to limit domestication are called integrated programs (Mobrand et al. 2005; Appleby et al. 2014). Integrated programs exist along a spectrum from almost 100% wild‐origin brood stock to only a few percent wild‐origin brood stock (Anderson et al. 2020). These strategies are not mutually exclusive, and hatchery programs will typically use different combinations of these two mitigation strategies to achieve their target level of impact on the wild population, depending on which measures are easier to implement (Appleby et al. 2014; Anderson et al. 2020). This creates a spectrum of possible mitigation strategies that can achieve the same overall theoretical level of genetic impacts on wild populations by controlling gene flow from the hatchery and introducing wild‐origin broodstock into the hatchery to varying degrees.

Deterministic models have shown that mitigation strategies along this spectrum can, if implemented perfectly, all have roughly equivalent genetic impacts on wild populations (Ford 2002; Appleby et al. 2014). For example, higher rates of gene flow from the hatchery to the wild can be offset by including a greater proportion of wild‐origin fish in the hatchery broodstock and vice versa. The models used to derive this relationship assume constant rates of gene flow between the hatchery and wild population (Ford 2002, Appleby et al. 2014), but in practice, these rates might vary over time due to changes in relative abundances of hatchery and wild‐origin fish, or the programs might miss their long‐term targets due to imperfect implementation. This variation in gene flow around the target rates creates a risk that the genetic impacts of the hatchery on the wild population exceed management targets, inadvertently reducing the fitness of the wild population. Understanding how this risk depends on the type of mitigation strategy can help identify robust approaches to mitigating the fitness consequences of hatcheries, where robustness is defined as how well the mitigation strategy performs at maintaining the wild genotype close to its theoretically expected value.

Here, we quantify whether and how the risk from implementation error depends on the degree to which a hatchery program emphasizes an integrated versus segregated approach. To this end, we developed both a deterministic and a stochastic population genetic model based closely on Ford (2002) in which the proportion of hatchery spawners in the wild (pHOS) and the proportion of wild‐origin broodstock (pNOB) vary over time. We used the deterministic model to test how robust different mitigation strategies were to small but long‐term deviations in the rate of gene flow from the management target. We use the stochastic model to test the extent to which variability in the relative abundances of hatchery and wild fish results in variation in the average genotypes and fitness of wild populations. We used these two models to compare the robustness of alternative mitigation strategies to implementation error along a spectrum of integration levels (pNOB between zero and one). This theoretical analysis tests how robust each mitigation strategy is, holding the level of implementation error constant. However, in practice, the level of implementation error may vary systematically between different mitigation strategies. To account for this possibility, we tested how alternative mitigation strategies performed under empirically observed levels of implementation error by parametrizing the stochastic population genetic model using demographic data from salmon and steelhead hatchery programs in Washington and Oregon.

## 2 Methods

### 2.1 Model Overview

**FIGURE 1:**
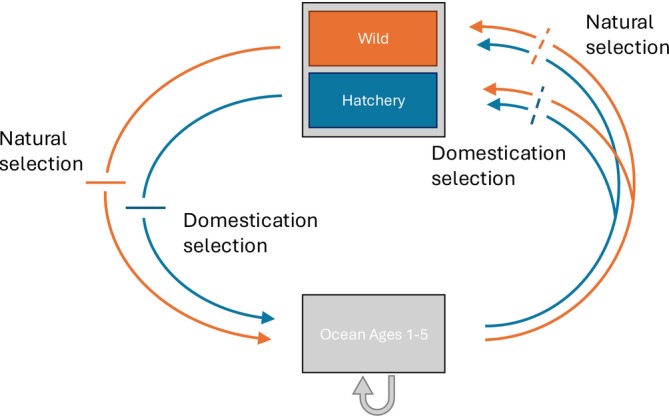
Schematic diagram of the population genetic model. The blue and orange arrows show the life cycle of hatchery‐ and wild‐origin fish, respectively. Hatchery‐ and wild‐origin fish spend 1–5 years in the ocean. Their maturation schedule determines the relative abundance of each age cohort when spawning. We assume mature individuals either spawn in the hatchery or in the wild, depending on the hatchery's management strategy and the degree of implementation error. We consider two alternative scenarios for selection. In the first scenario, selection acts early in the life cycle, and natural selection only acts on wild‐origin fish and domestication selection only acts on hatchery‐origin fish (solid lines). In the second scenario, selection occurs after individuals have returned to spawn, and both wild‐ and hatchery‐origin fish spawning in the hatchery experience domestication selection and wild‐ and hatchery‐origin fish spawning in the wild experience natural selection (dashed lines).

We developed a quantitative genetic model based closely on Ford (2002) that describes the genetic state of a salmon population with some individuals reproducing in the wild and some in a hatchery (Figure 1). The naturally reproducing portion of the population experiences natural selection, and the hatchery‐reared portion of the population experiences domestication selection. We modeled the effect of these selection events on a single quantitative trait where domestication selection favors larger values of the trait. We extended this quantitative genetic framework to incorporate the age structure of the population and to allow the relative abundance of the hatchery and natural individuals to fluctuate over time. We then tested the influence of variability in the relative abundance of hatchery‐ and natural‐origin fish on the genetic state of each portion of the population.

The goal of our analysis is to compare alternative management strategies for mitigating domestication in terms of their robustness to demographic variability and implementation error. To quantify this relationship, we constructed alternative management strategies that achieve the same expected level of impact on the wild population through different combinations of the two primary mitigation strategies, limiting gene flow from the hatchery and incorporating natural‐origin fish into the broodstock. We defined each management strategy by first setting the target level of genetic impact, which we quantified using the proportion of natural influence statistic (PNI, defined in detail in Section 2.3) that describes how close the genotypes of the wild population are to the optimum under natural selection (HSRG 2009). Next, we set the pNOB to a value between zero and one. From the target PNI and the value of pNOB, we use the population genetic model to solve for the target level of pHOS that will achieve the target PNI.

We then used the population genetic model to quantify how robust each of these combinations of pNOB and pHOS is to different forms of implementation errors (Table 1). We tested the influence of systematic, long‐term deviations from the target values of pHOS and pNOB using a local sensitivity analysis and then used simulations to test the effects of stochastic fluctuations around the average values of pHOS and pNOB. We also used simulations to test how robust each mitigation strategy was to rare events that cause a large increase in the rate of gene flow from the hatchery for a short time. Finally, we compared these results to empirically observed levels of implementation error estimated using demographic data from hatchery programs in Washington and Oregon.

**TABLE 1:** List of analyses and research questions.

Section	Analysis	Research question	Approaches	Figure
2.6.1, 2.6.4	Stochastic implementation errors	How robust are the alternative hatchery management strategies to variation in pHOS pNOB over time, as we might expect to see given inter‐annual variation in population abundance?	Holding the standard deviation of pHOS and pNOB constant between scenarios	Figure 4, Figure 5
			Holding the coefficient of variation of pHOS and pNOB constant between scenarios	Figure 5
			Empirically observed variation in pHOS and pNOB	Figure 7
2.6.2	Individual large events	How robust are the alternative management strategies to individual years with large deviations from target values? For example, if measures like weirs flood and fail to prevent hatchery‐origin fish from spawning in the wild.		Figure 5
2.6.3	Long‐term implementation errors	How robust are alternative management strategies to small but biased deviations from management targets? For example, if estimates of pHOS are systematically biased?	Deviations around the management target: measured in absolute terms	Figure 6
			Deviations from management targets: measured as a percentage of the target	Figure 6

### 2.2 Population Genetic Model

We developed a dynamic population genetic model that tracks the distribution of a quantitative trait in a hatchery and wild salmon population. The model tracks the mean $g_{a,i,t}$and variance $V_{a,i,t}$of the genetic distribution of fish of age $a\in \left\{0,1,2,3,4,5\right\}$ and of origin $i\in \left\{W:\text{wild},H:\text{hatchery}\right\}$ in year t through two demographic events: reproduction and selection. In the reproductive step, the contribution of each age class is determined by their relative abundances $N_{a}$ and fecundities $F_{a}$, which we assume are constant over time and reflect the rates of maturation of each cohort during their marine life phase (Figure 1). The relative contribution of hatchery and wild‐origin fish is determined by the relative abundance $p_{i,j,t}$ of hatchery‐origin $\left(i=H\right)$and wild‐origin fish $\left(i=W\right)$in each environment ($j\in \left\{H,W\right\}$). The proportion of hatchery fish spawning in the wild $p_{W,H,t}$ corresponds to pHOS, and the proportion of wild fish spawning in the hatchery $p_{H,W,t}$ corresponds to pNOB.

The mean genotype after reproduction in the hatchery is determined by the average genotypes $g_{a,i,t}$in each age class of hatchery and wild‐origin fish, weighted by their relative abundances $N_{a}$and fecundity at age $F_{a}$, divided by a normalizing constant $Z_{H}$

(1) $$g_{0,H,t}=\frac{1}{Z_{H}}\sum_{i\in \left\{H,W\right\}}\sum_{a=1}^{A}p_{i,H,t}F_{a}N_{a}g_{a,i,t}.$$

The same formula applies to the reproduction in the wild, except we assume the reproductive success of hatchery‐origin fish is lower by a factor ϕ due to the environmental effects of captive rearing

(2) $$g_{0,W,t}=\frac{1}{Z_{W}}\sum_{i\in \left\{H,W\right\}}\sum_{a=1}^{A}p_{i,W,t}\phi^{I_{i=H}}F_{a}N_{a}g_{a,i,t}.$$

The normalizing factor $Z_{W}$ is the total fecundity of the population and $I_{i=H}$ is an indicator variable equal to one for hatchery‐origin fish and zero for wild‐origin fish.

The genetic variance of each new cohort $V_{0,i,t}$ is determined by the genetic variance in the spawning population $V_{\text{spawn},i,t}$ and the recombination variance $V_{r}$ (the amount of genetic variance among siblings). Following Turelli and Barton (1994), we approximate the genetic distribution of each cohort with a normal distribution. Using this approximation, the genetic distribution of the spawning population is a mixture of normal distributions. The variance of a mixture of normal distributions is the weighted average of the variances of each component plus the variance of the component means. Applying this formula to the wild population yields

(3) $$\begin{array}{c} V_{\text{spawn},W,t}=\frac{1}{Z_{W}}\sum_{i\in \left\{H,W\right\}}\sum_{a=1}^{A}p_{i,W,t}\phi^{I_{j=H}}F_{a}N_{a}V_{a,i,t}\\ \;+\frac{1}{Z_{W}}\sum_{i\in \left\{H,W\right\}}\sum_{a=1}^{A}\left[p_{i,W,t}\phi^{I_{j=H}}F_{a}N_{a}g_{a,i,t}^{2}-g_{0,W,t}^{2}\right]. \end{array}$$

The formula for the hatchery population would be the same, except it would omit the factor $\phi^{I_{i=H}}$, which accounts for the environmental effect of captive breeding on reproductive success. Assuming mate selection is random, the genetic variance in the new cohort after reproduction is equal to one‐half the variance of the spawning population plus the recombination variance

(4) $$V_{0,i,t}=\frac{1}{2}V_{\text{spawn},i,t}+V_{r}.$$

Following Ford (2002), both hatchery and wild populations experience stabilizing viability selection around an optimum phenotype $\theta_{i}$ that differs between the two environments. The probability of survival is determined by an individual's phenotype and the variance of the phenotype‐based selection function $\sigma_{p,i}^{2}$, which is inversely related to selection strength. However, our model tracks genotypes, so we derive the probability of survival $s_{i}$ of an individual of origin i with genotype g

(5) $$s_{i}\left(g\right)=s_{\max}e^{-\frac{1}{2}{\left(\frac{g-\theta_{i}}{\sigma_{i}}\right)}^{2}}$$

where the maximum survival rate $s_{\max}$ and the genotype‐based selection variance $\sigma_{i}^{2}$ are determined by the heritability of the trait and the phenotype‐based selection variance $\sigma_{i}^{2}$ (we derive Equation (5) based on a model of selection acting on phenotypes in Appendix S1). The mean genotype of the cohort after selection is determined by the genetic variance $V_{0,i,t}$, the selection variance $\sigma_{i}$, and the optimum phenotype $\theta_{i}$

(6) $$g_{0,i,t}^{\prime }={\left(\frac{1}{V_{0,i,t}}+\frac{1}{\sigma_{i}^{2}}\;\right)}^{-1}\left(\frac{g_{0,i,t}}{V_{0,i,t}}+\frac{\theta_{i}}{\sigma_{i}^{2}}\;\right).$$

The genotype variance after selection depends on the selection variance

(7) $$V_{0,i,t}^{\prime }={\left(\frac{1}{V_{0,i,t}}+\frac{1}{\sigma_{i}^{2}}\;\right)}^{-1}.$$

We derive Equations (6 and 7) in Appendix S2.

We tested two scenarios for the timing of selection in the life cycle: viability selection acting early in the life cycle, and fecundity selection acting at the time of reproduction. We assume viability selection acts early in the life cycle; therefore, wild‐origin fish experience natural selection, and hatchery‐origin fish experience domestication selection. In contrast, fecundity selection acts at reproduction; therefore, the individuals spawning in the hatchery experience domestication selection regardless of their origin and vice versa. In this alternative scenario, Equations (6 and 7) are applied to the genotype distribution in each cohort before reproduction.

### 2.3 Model Parameters

We provide all parameters in Table 2 and detail our parameterization process below.

**TABLE 2:** Population genetic model parameters.

Parameter	Interpretation	Value	Source(s)
A	The maximum age class included in the model	Chinook: 5 Steelhead: 5 Coho: 4 Pink: 2	(Healey 1991; Sandercock 1991; Johnson 2004)
$F_{a}$	Fecundity at age, relative to the maximum fecundity at age	Chinook: $\left\{0.03,0.28,0.58,0.83,1\right\}$ Chinook b = 2: $\left\{0.09,0.42,0.70,0.88,1\right\}$ Chinook b = 4: $\left\{0.01,0.19,0.49,0.78,1\right\}$ Steelhead: $\left\{0.30,0.51,0.56,0.93,1\right\}$ Coho: $\left\{0,0.21,1,0.98,0.0\right\}$ Pink: $\left\{0,1,0,0,0\right\}$	(Johnson 2004, Ohlberger et al. 2020)
$N_{a}$	Relative abundance of spawners	Chinook: $\left\{0,0.14,0.43,0.40,0.03\right\}$ ^a^ Steelhead: $\left\{0,0.01,0.54,0.42,0.02\right\}$ Coho: $\left\{0,0.18,0.73,0.10,0.0\right\}$ Pink: $\left\{0,1,0,0,0\right\}$	(Healey 1991, Sandercock 1991, Johnson 2004)
$\mathrm{RR}S^{*}$	Fitness tradeoff between hatchery and natural environment	0.3^a^ (0.1, 0.5, 0.7)	(Araki et al. 2007, Ford et al. 2016, Koch and Narum 2021)^b^
$\sigma_{i}^{2}$	Variance of fitness function around optimum phenotype	20 (10,100)^c^	(Ford 2002, Baskett and Waples 2013)
$V_{r}$	Recombination variance	0.5 (w.l.o.g.)^d^	
$\theta_{W}$	Optimum phenotype wild	0.0 (w.l.o.g.)	
$\theta_{H}$	Optimum phenotype hatchery	7.1^a^ (5.1, 15.6, 3.9, 2.8)	Determined by RRS and $\sigma_{i}^{2}$
$\tau_{W,H,t}$	Standard deviation of variability in pNOB	0.0^a^ (0.0, 0.25)^e^	Section 2.6.4, Table S3
$\tau_{H,W,t}$	Standard deviation of variability in pHOS	0.15^a^ (0.0, 0.25)^e^	Section 2.6.4, Table S3
$\rho_{W,H,t}$	Autocorrelation in pNOB	0.25^a^ (0.25–0.9)^e^	Section 2.6.4, Table S3
$\rho_{H,W,t}$	Autocorrelation in pHOS	0.0^a^ (0.25–0.9)^e^	Section 2.6.4, Table S3

^a^ Parameter values used for simulations in Figure 4.

^b^ This article reviews many studies measuring life‐time reproductive success across species and hatchery management practices. Our parameter choices are based on their analysis of studies that compared reproductive success between wild and hatchery origin fish that have been raised in captivity for many generations (e.g., Ford et al. 2016).

^c^ These values are hard to measure experimentally, so we chose a range that is consistent with prior modeling studies.

^d^ Without loss of generality, that is, we need to choose a specific value for a calculation, but even though the specific choice does affect the outcome.

^e^ In our simulation analyses, we test the sensitivity of the model to the variance τ and autocorrelation. ρ parameters, so a wide range of values is used. We devote a full analysis to estimating specific values for these parameters from population time series data.

#### 2.3.1 Age Distribution of Spawners

The relative abundance of each age class $N_{a}$ was chosen to match common age distributions of four species of Pacific salmon: Chinook, coho, steelhead, and pink. The age distributions of each species were chosen to match the data from spawning surveys in the Columbia River and its tributaries in 2014, 2015, and 2016, accessed through the RMIS database (Johnson 2004). We calculated age distributions for fall run Chinook salmon, summer run steelhead, and late fall (type N) coho by summing the total number of individuals observed in a spawning survey from each species and age class (see Appendix S3 for details). For pink salmon, we assumed all individuals spawn at age two. These age distributions are fairly representative of Chinook and coho salmon throughout their range, although there is considerable variation (Healey 1991; Sandercock 1991).

#### 2.3.2 Fecundity at Age

We used length‐at‐age data from spawning surveys reported in the RMIS database for the afore mentioned species and run type groups to calculate the fecundity at age parameters $F_{a}.$For each fish reported in the spawning surveys we calculated its expected fecundity using an allometric relationship ($F=\alpha L^{b}$). Specific values for the parameters of this relationship are not well documented in our study region (Myers et al. 1998). However, studies in other regions have shown that scaling parameters b vary between 2 and 4.5, depending on the measure of fecundity (e.g., number of eggs, total egg mass; Ohlberger et al. 2020). As a base assumption, we used a scaling parameter b=3and tested the sensitivity of our results to this parameter by calculating fecundity at age for Chinook salmon with b=2 and b=4. The proportionality constant αdoes not influence our results because the model only tracks the relative contributions to reproduction of each age class, so we calibrated this value to set the maximum fecundity at age equal to one. Once we calculated the estimated fecundity for every observation in the sample, we grouped the observations by age and species to determine the average fecundity at age (detailed calculations given in Appendix S4).

#### 2.3.3 Population Genetic Parameters

We calibrated the parameters of our genetic model to match empirical estimates of the long‐term fitness tradeoffs between wild and hatchery environments (Koch and Narum 2021). We calculated fitness tradeoffs by comparing the average fitness of a population fully adapted to the natural environment to a population fully adapted to the hatchery, which we call $\mathrm{RR}S^{*}$, the equilibrium relative reproductive success of hatchery fish after many generations in captivity. We calculate this quantity by first computing the equilibrium genetic mean $g_{H}^{*}$and variance $V_{H}^{*}$ of a population only raised in captivity for many generations (pNOB=0). We then calculated the genetic mean $g_{W}^{*}$and variance $V_{W}^{*}$ in an undisturbed wild population (pHOS=0). Finally, we compared the fitness of these two populations under natural selection

(8) $$\mathrm{RR}S^{*}=\frac{\int_{-\infty }^{\infty }e^{-\frac{{\left(g-g_{H}^{*}\right)}^{2}}{2V_{H}^{*}}}e^{-\frac{\left(g-\theta_{W}\right)}{2\sigma_{H}^{2}}}\mathrm{dg}}{\int_{-\infty }^{\infty }e^{-\frac{{\left(g-g_{W}^{*}\right)}^{2}}{2V_{W}^{*}}}e^{-\frac{\left(g-\theta_{W}\right)}{2\sigma_{W}^{2}}}\mathrm{dg}}.$$

Smaller values of $\mathrm{RR}S^{*}$ imply greater fitness tradeoffs between wild and hatchery environments. Note that $\mathrm{RR}S^{*}$ does not necessarily reflect the relative reproductive success of wild and hatchery populations in our simulations because it refers to the limiting case where both populations are fully adapted to their respective environments. Furthermore, the fitness‐based $\mathrm{RR}S^{*}$ parameter is distinct from the parameter ϕ in Equation (3), which accounts for non‐genetic environmental effects of the hatchery on reproductive success.

### 2.4 Modeling Mitigation Strategies

**FIGURE 2:**
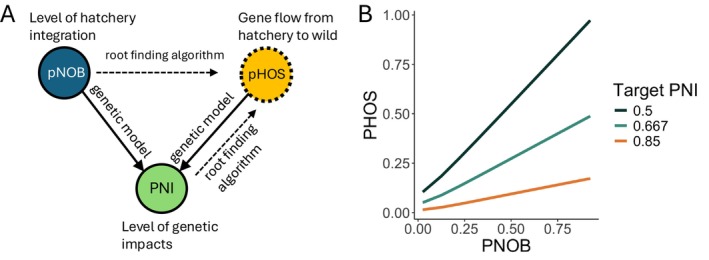
The relationships between the three quantities that define the mitigation strategies explored in our analysis: the proportion of natural origin brood stock (pNOB), the proportion of hatchery‐origin spawners (pHOS), and the level of domestication of the wild population, quantified by proportion of natural influence (PNI). (A) The values of pNOB and pHOS determine the level of domestication, PNI (solid arrows). A management strategy is defined by target values of pNOB and PNI (solid outlines). The target value of pNOB determines where the management strategy falls along the spectrum from segregated programs (pNOB = 0) to fully integrated programs (pNOB = 1.0). The target value of PNI determines the expected theoretical level of genetic impact through domestication selection that the hatchery will have on the wild population. We solve for the target value of pHOS (dotted arrows) that achieves the target value of PNI given the specified value of pNOB. (B) The genetic impact of the hatchery can be mitigated by either reducing pHOS or increasing pNOB, which creates a positive relationship between pHOS and pNOB for a given level of PNI. We compare management strategies that target values of pHOS and pNOB falling along the three lines in panel B.

We quantified the relationship between the management strategy and robustness to imperfect implementation by constructing management scenarios that, if implemented perfectly, would lead to the same average level of genetic impacts (Figure 2). We quantified the level of genetic impacts from the hatchery on the wild population using the proportionate natural influence (PNI) statistic. The statistic was developed by the Hatchery Scientific Review Group (HSRG 2009) and is defined as the equilibrium genotype of the wild population $g_{w}^{*}$ assuming constant values of pHOS and pNOB scaled so that it takes the value of one when the genotypes are equal to the optimum genotype under natural selection $\theta_{W}$ and zero when they are equal to the optimum genotype under domestication selection $\;\theta_{H}$.

(9) $$\mathrm{PNI}\left(\text{pNOB},\text{pHOS}\right)=\frac{g_{w}^{*}\left(\text{pNOB},\text{pHOS}\right)-\theta_{H}}{\theta_{W}-\theta_{H}}.$$

Note that we write the equilibrium genotype $g_{w}^{*}$as a function of pHOS and pNOB to emphasize that its value depends on these two quantities (Figure 2A). The HSRG (2009) derived an approximation of this quantity from the model presented in Ford (2002) that is a function of pHOS and pNOB

(10) $$\mathrm{PNI}\left(\text{pNOB},\text{pHOS}\right)\approx \frac{\text{pNOB}}{\text{pHOS}+\text{pNOB}}.$$

However, this approximation does not account for differences between the relative abundances (pHOS and pNOB) and the actual rates of gene flow. To account for these effects, we use the exact value of PNI found by computing the equilibrium genotype from our population genetic model. We chose to construct scenarios with target PNI values of 0.5, 0.667, and 0.85. The two smaller values correspond with published hatchery management guidelines (HSRG 2009; Appleby et al. 2014; Withler et al. 2018; Anderson et al. 2020), and the third represents a more precautionary alternative.

We compared mitigation strategies that achieved these target levels of PNI, $\bar{\mathrm{PNI},}$with target levels of pNOB, $\bar{\text{pNOB}},$ between zero and one. For a given values of $\bar{\mathrm{PNI}}$ and $\bar{\text{pNOB}}$ we solved for the target value of pHOS, $\bar{\text{pHOS},}$ by minimizing the difference between the target value of $\bar{\mathrm{PNI}}$ and the value predicted by the model $\mathrm{PNI}\left(\bar{\text{pNOB}},\text{pHOS}\right)$

(11) $$\bar{\text{pHOS}}={\text{argzero}}_{\text{pHOS}}\left(\bar{\mathrm{PNI}}-\mathrm{PNI}\left(\bar{\text{pNOB}},\text{pHOS}\right)\right).$$

We solved the root finding problem in Equation (10) using the bisection search algorithm. In general, smaller values of $\bar{\text{pNOB}}$ require smaller values of $\bar{\text{pHOS}}$ to achieve a target value of $\bar{\mathrm{PNI}}$ (Figure 2B).

### 2.5 Quantifying Genetic Impacts on the Wild Population

We quantified the effect of the hatchery on the genetic state of the wild population using a metric derived from the models, which we call the domestication index. This metric measures changes in the average genotype of the wild population $g_{0,W,t}$scaled to equal zero when $g_{0,W,t}$ is equal to the optimum phenotype under natural selection and one when $g_{0,W,t}$is equal to the optimum under domestication selection.

(12) $$DI_{t}=\frac{g_{0,W,t}-\theta_{W}}{\theta_{H}-\theta_{W}}.$$

This metric allows us to quantify changes in genotypes in terms of their ecological significance (fitness in each environment) across scenarios with different genotype distributions and selection functions. The proportionate natural influence and domestication index statistics are related by PNI=1−DI. We have chosen to report the result of variability in genotype over time in terms of the domestication index rather than PNI, because PNI is typically used to describe long‐term outcomes, and we track both the long‐term average values and variation in genotypes over time in our simulations. Furthermore, larger values DI correspond with larger impacts on the wild population, which makes the results easier to follow. However, note that the domestication index normalizes the level of genotype change as a function of the differences in selective environments $\left(\theta_{H}-\theta_{W}\right)$. It has a maximum value of one (fully adapted to the hatchery) and zero (fully adapted to the wild). As a result, the biological significance of a change in the domestication index will depend on the strength of the fitness tradeoff between the hatchery and the wild. If the fitness tradeoffs ($\mathrm{RR}S^{*}$) are small, then a large domestication index might not have a large impact on the wild population, and if the fitness tradeoff is large, even small values of the domestication index could have significant impacts.

### 2.6 Modeling Implementation Errors

We tested the effects of deviations from the target values of pHOS and pNOB specified by the mitigation strategies on the genotypes of the wild population across the management scenarios. We constructed four scenarios to test how the sensitivity of the wild population depends on the functional form and time scale of these deviations: (1) stochastic fluctuations around the average value, (2) single years with large deviations, (3) long‐term directional changes in the average value, and (4) empirically observed levels of demographic variability (Table 1).

#### 2.6.1 Stochastic Demographic Variability

Implementation errors can take the form of short‐term fluctuations around the management targets. We modeled the effects of short‐term fluctuations by simulating the relative abundances of the hatchery and wild populations as a stochastic process. The relative abundance $p_{W,H,t}\equiv \mathrm{pNO}B_{t}$and $p_{H,W,t}\equiv \mathrm{pHO}S_{t}$must be between zero and one, and to satisfy this constraint we modeled changes in these quantities with a log‐odds transformed auto regressive process, where $\text{logit}\left(x_{i,j,t}\right)=p_{i,j,t}$ and $x_{i,j,t}$ follows an AR1 process with means $\mu_{i,j}$, variances $\tau_{i,j}^{2}$ and autocorrelations coefficients $\rho_{i,j}$

(13) $$p_{i,j,t}=\frac{1}{1+e^{-x_{i,j,t}}}$$

(14) $$x_{i,j,t}=\rho_{i,j}x_{i,j,t-1}+\frac{\epsilon_{i,j,t}}{1-\rho_{i,j}\;}$$

(15) $$\epsilon_{i,j,t}∼N\left(\mu_{i,j},\tau_{i,j}^{2}\right).$$

We tuned the values of the mean $\mu_{i,j}$and variance $\tau_{i,j}^{2}$parameters to achieve target values for the expectation $E\left[p_{i,j,t}\right]$ and variance $V\left[p_{i,j,t}\right]$ of the relative abundances used in the simulation experiments. We varied the autocorrelation parameter $\rho_{i,j}$ to test the sensitivity of our findings to the time scale of the implementation errors.

We quantified the effects of this demographic variability on genetic outcomes by calculating the variation of the domestication index over time $V\left[DI_{t}\right]$. We simulated the dynamics of the genetic model by sampling the relative abundances $p_{i,j,t}$ at each time step from Equations ((12), (13), (14)). We then recursively applied Equations ((1), (2), (3), (4), (5), (6)) to update the genetic state of the population. We initialized simulations with each population adapted to the natural environment $g_{a,i,t}=\theta_{W}$and ran the simulation for 20,000 time‐steps. We removed the first 500 to eliminate the influence of initial conditions, and we used the final 19,500 to calculate the mean and variance of the domestication index.

We tested how the variance of the domestication index changed across mitigation strategies, holding the total variance of the relative abundances constant between scenarios and holding the coefficient of variation of the relative abundance constant between scenarios. Holding the coefficient of variation constant between scenarios assumes that the level of implementation errors has a one‐to‐one relationship with the target value (smaller targets = smaller errors). Holding the variance constant implies that the scale of the target and the magnitude of implementation errors are independent.

#### 2.6.2 Large Low Probability Events

Implementation errors can also take the form of a single large deviation from target values. We modeled this scenario by simulating the change in domestication caused by a single year where pHOS shifted from the management target to 100%. While this simulated change may be unrealistically large, it allows us to evaluate the performance of the strategies under extreme conditions, while the other analyses ensure our findings remain empirically grounded. We simulated these scenarios by running the model for 500 steps to reach equilibrium and then simulating 1 year with. We then simulated the model for another 5 years after the event to allow the genetic effects of the event to propagate through the population's age structure, at which point we calculated the domestication index.

#### 2.6.3 Long‐Term Implementation Errors

We evaluated the effects of long‐term deviations from demographic targets using a local sensitivity analysis, which characterizes the effect of a small change in a parameter on an outcome variable. In this case, we tested the effects of a change in the long‐term average level of pHOS and pNOB on the domestication index. We evaluated this outcome by simulating the dynamics of the genetic model (Equations (1), (2), (3), (4), (5), (6), (7)) holding the relative abundances of the hatchery and wild populations constant over time at the target values $\bar{\text{pNOB}}$ and $\bar{\text{pHOS}}$ for 500 time steps, until genotypes of the hatchery and wild population reached equilibrium.

We used the finite difference method to calculate the sensitivity of the domestication index to small changes in the long‐term average values of pHOS and pNOB. The sensitivity $S_{\phi }^{\mathrm{DI}}=\frac{\mathrm{\partial DI}}{\partial \phi }$ is defined as the partial derivative of the outcome DI with respect to the parameter $\phi \in \left\{\text{pHOS},\text{pNOB}\right\}$. We also calculated the elasticity of the domestication index $E_{\phi }^{\mathrm{DI}}=\phi \frac{\mathrm{\partial DI}}{\partial \phi }$, which represents the effects of a proportional change in the parameter ϕ on the outcome.

#### 2.6.4 Empirically Observed Demographic Variability

**FIGURE 3:**
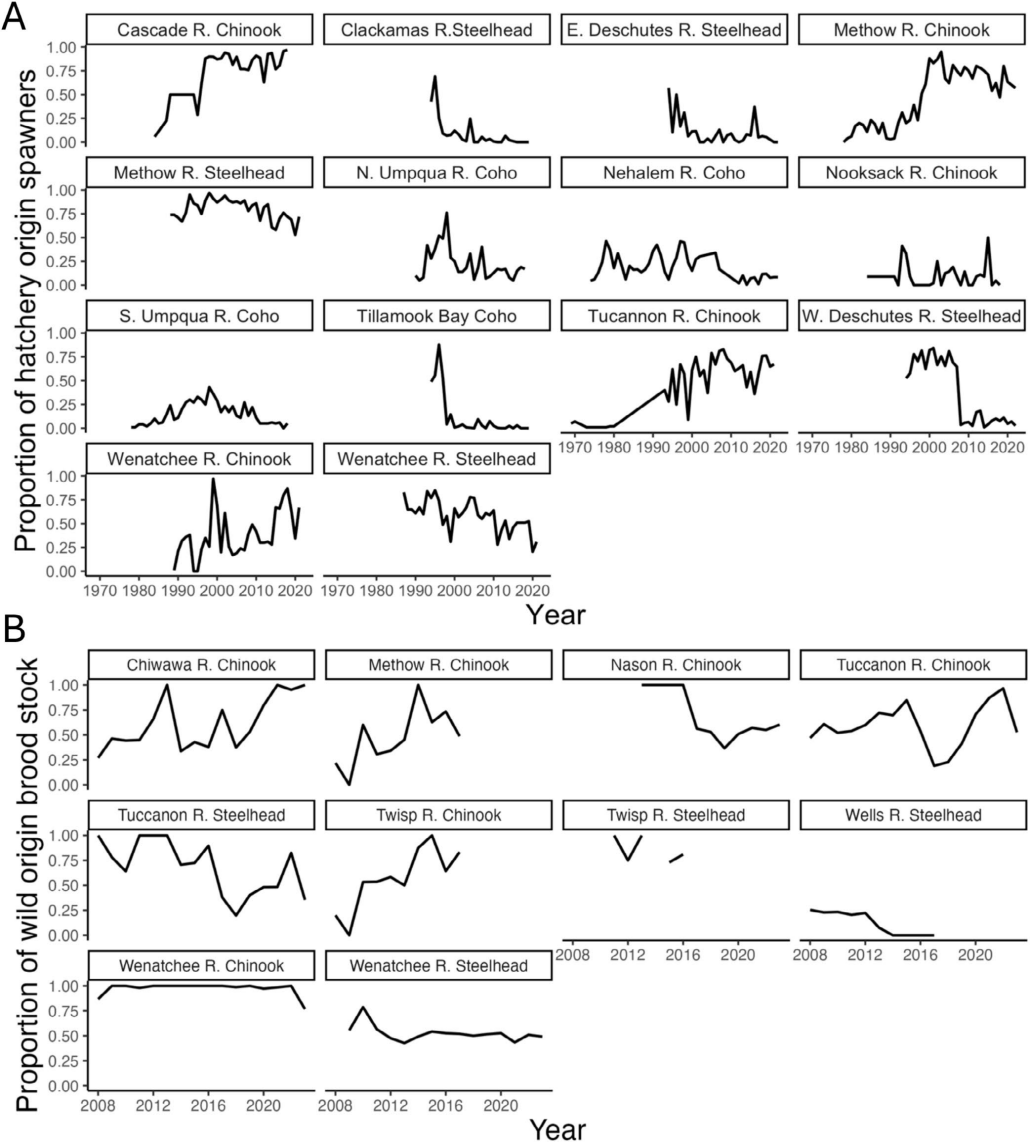
Time series of (A) pHOS and (B) pNOB from hatchery monitoring programs in Oregon and Washington. The pairs of pHOS and pNOB data used to parameterize our simulations are given in Table S1.

We estimated the empirical levels of demographic variability from a sample of salmon and steelhead hatchery programs in Washington and Oregon (Figure 3). We used these estimates to construct management scenarios consistent with empirically observed levels of demographic variability in integrated and segregated hatchery programs by estimating the mean, variance and autocorrelation of ${\text{pHOS}}_{t}$and ${\text{pNOB}}_{t}$ time series associated with each hatchery program. We then simulated the population genetic model, sampling ${\text{pHOS}}_{t}$and ${\text{pNOB}}_{t}$ at each time step from Equations ((13), (14), (15)). We parameterized the model to match the estimated means $\mu_{i,j}$, variances $\tau_{i,j}^{2}$, and autocorrelation $\rho_{i,j}$for each population. We used these simulations to predict how much the of observed level of demographic variation resulted in variability in the domestication index.

We used data on spawning abundances collected by a variety of state, federal, tribal, and local agencies to estimate pHOS for each hatchery program. The data were compiled in the most recent National Marine Fisheries Service status review report. See Ford (2022) for details. The agencies operating salmon hatcheries collect and retain information on pNOB, but there is currently no single regional source for these data. For the segregated hatchery programs included in our sample, we checked the Hatchery Genetic Management Plans to verify that no wild‐origin fish were intentionally incorporated in the hatchery brood stock during the sampled period (Oregon Department of Fish and Wildlife 2025). For the integrated hatchery programs, we used pNOB information provided by the Washington Department of Fish and Wildlife (K. Warheit, pers. comm). The data we obtained included both segregated and integrated hatchery programs for Chinook and steelhead and segregated hatchery programs but not integrated programs for coho; as a result we only ran simulations of segregated hatchery programs with coho salmon. We also were unable to obtain data for pink salmon hatcheries.

For several hatchery programs, the pHOS data were reported at a larger spatial scale than the pNOB data. For example, pHOS data were reported for the entire Wenatchee River basin, which includes several hatchery programs with independent pNOB time series. In these cases, we matched the pNOB data from the hatchery to the closest available pHOS data to construct a parameter set for our simulation analysis. These combinations of pNOB and pHOS data used in our simulation analysis are given in Table S1. A list of segregated hatchery programs is shown in Table S2 and the corresponding estimates of the mean, variance and autocorrelation parameters for pHOS and pNOB are given in Tables S3 and S4.

We used Bayesian time series models to estimate the demographic variability parameters for each population and hatchery program included in our dataset. The models estimated the total variance of the time series along with the presence of autocorrelation and long‐term trends. Although we do not include trends in our simulation, we included this term in the estimation models because if a trend is present in the data, it would inflate our estimates of autocorrelation.

We used a hierarchical model structure to increase the precision of the parameter estimates. Hierarchical models apply to datasets that contain multiple related groups. In our case, each time series from an individual hatchery program or population constitutes a group. For each time series i our model computes the mean $\mu_{i}$, variance $\tau_{i}$, trends $\beta_{i}$ and autocorrelation $\rho_{i}$. In a standard Bayesian model, we would specify a unique prior distribution over the parameters for each time series. Hierarchical models estimate the prior distribution based on the observed differences between groups. By estimating the prior distribution, hierarchical models pool information between groups, thereby increasing the precision of the parameter estimates. We fit the model with a no‐U‐Turns sampling algorithm implemented with stan modeling software (Stan Development Team 2020). The details of the model structure are given in Appendix S5, priors for the hierarchical model are listed in Table S5 and parameters used for the no‐U‐Turns algorithm are listed in Table S6.

## 3 Results

### 3.1 Stochastic Demographic Variability

**FIGURE 4:**
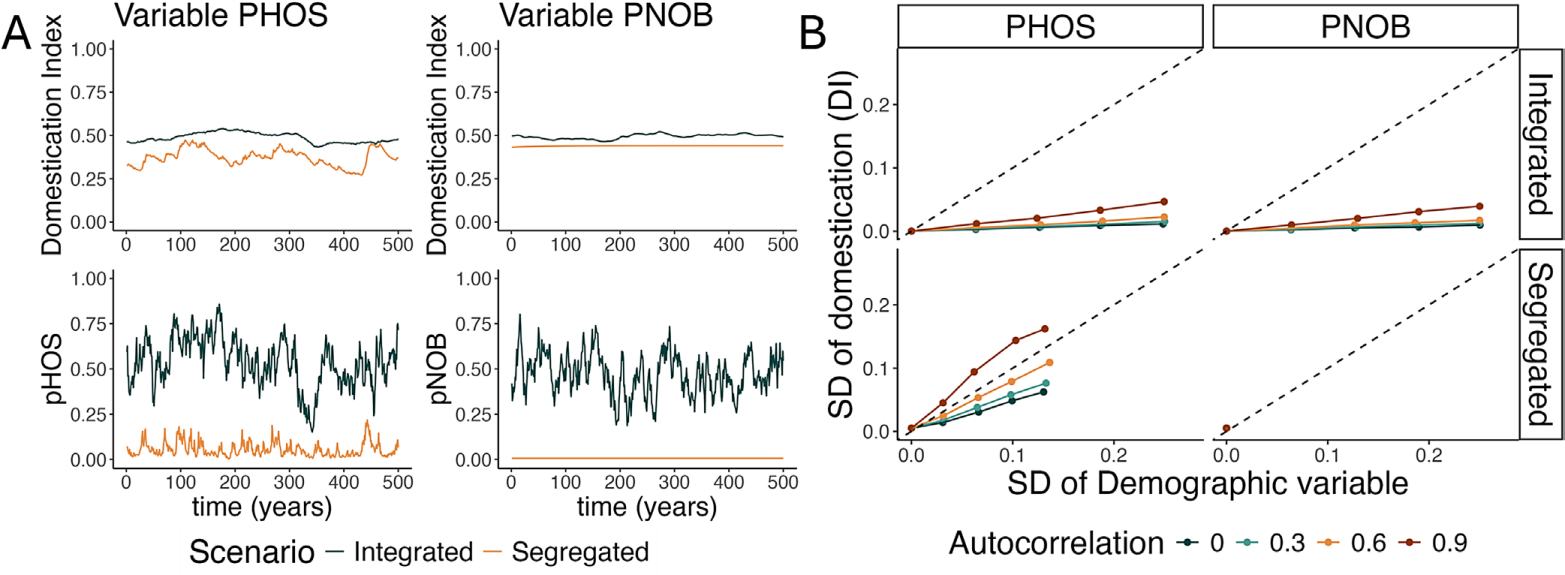
The effects of variation in the proportions of hatchery‐origin spawners (pHOS) and wild‐origin brood stock (pNOB) on the average genotype of the wild population. We quantify the changes in genotypes using the domestication index, which takes the value of one when wild‐origin fish are perfectly adapted to the hatchery and zero when they are adapted to the natural environment. (A) An example time series showing the effect of varying pHOS and pNOB on the domestication index (DI) in a segregated (orange lines) and integrated (blue lines) hatchery. (B) The effects of the variance and autocorrelation of pHOS and pNOB on the variation of the domestication index. Note that the variability of pNOB does not apply to segregated programs that do not incorporate wild‐origin brood stock. For the example simulations (panel A), the standard deviation of pHOS and pNOB $\left(\tau_{H,W},\tau_{W,H}\right)$ were set to one‐quarter times the maximum variance given the average values of pHOS and pNOB, and the autocreation parameters $\left(\rho_{H,W},\rho_{W,H}\right)$ were set to 0.8.

Variation in the relative abundance of wild‐ and hatchery‐origin fish results in variability in the level of domestication of the impacted wild population (Figure 4A). Variation in the pHOS produced more variation in domestication for segregated than integrated programs (Figure 4A,B). Variation in pNOB caused variation in the level of domestication when the hatchery program had an integrated design, but does not affect segregated programs where pNOB = 0 (Figure 4). Variation in both pHOS and pNOB impacts the level of domestication of the wild population for integrated programs, but the effect is small compared to the effect of pHOS on segregated programs (Figure 4B). Finally, autocorrelated demographic variability has a much larger effect on the domestication of the wild population, because the corresponding changes in the rates of gene flow are more likely to be repeated sequentially allowing more time for its effects to build up in the population (Figure 4B).

**FIGURE 5:**
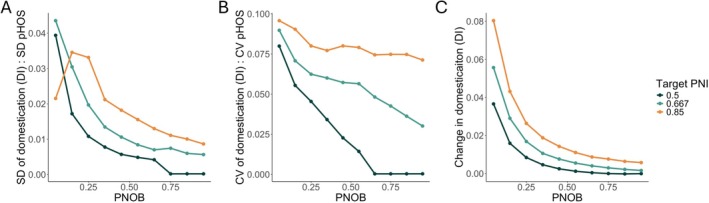
The effect of variation in the proportion of hatchery‐origin spawners (pHOS) on the variability of the domestication index as a function of the hatchery's integration level (average proportion of hatchery origin spawners). In (panel A), the standard deviation of pHOS is held constant between scenarios, while in (panel B), the coefficient of variation in pHOS is held constant between scenarios. (Panel C) shows the change in the domestication index after 1 year, where pHOS = 100%.

The relationship between the level of hatchery integration (target pNOB) and the robustness of the ability to mitigate genetic impacts of that hatchery on the wild population depends on the character of the implementation errors and the target level of impacts (PNI, Figure 5). If the standard deviation of pHOS is held constant between scenarios, the corresponding variation in the domestication index decreases quickly as a function of the level of hatchery integration (Figure 5A). However, when the coefficient of variation of pHOS is held constant between scenarios (i.e., implementation errors scale proportionally with the management target), the level of integration (pNOB) has a smaller effect on robustness and depends more strongly on the target value of PNI (Figure 5B). Finally, integrated hatchery programs are much more robust than segregated programs to large but rare increases in the rate of gene flow from the hatchery (Figure 5C). These patterns are consistent across several alternative sets of biological parameters (including different values of selection strength, which can significantly alter the amount of gene flow between the hatchery and wild population; Baskett and Waples 2013) as well as different timings of selection in the life cycle (Figures S1–S3). However, when the coefficient of variation is held constant between scenarios (Figure S2) there are some parameter sets where the sensitivity of the mitigation strategy to demographic variation increases as a function of pNOB (Figure S3).

Counterintuitively, the level of variability of the domestication index is higher for larger target values of PNI, which is somewhat surprising because larger values of PNI are generally associated with fewer genetic impacts on wild populations. However, PNI determines the average level of change in genotypes, whereas Figure 5 shows the level of variability around the average. The average level of domestication of the wild population is smaller for larger values of PNI, but our simulation shows that it will deviate from that lower level more easily if management targets are missed. However, these results do not indicate that higher values of PNI are inferior to lower values for mitigating genetic impacts on wild populations. It does suggest that maintaining a very low level of domestication could require more precise implementation of the management strategy.

### 3.2 Long‐Term Implementation Errors

**FIGURE 6:**
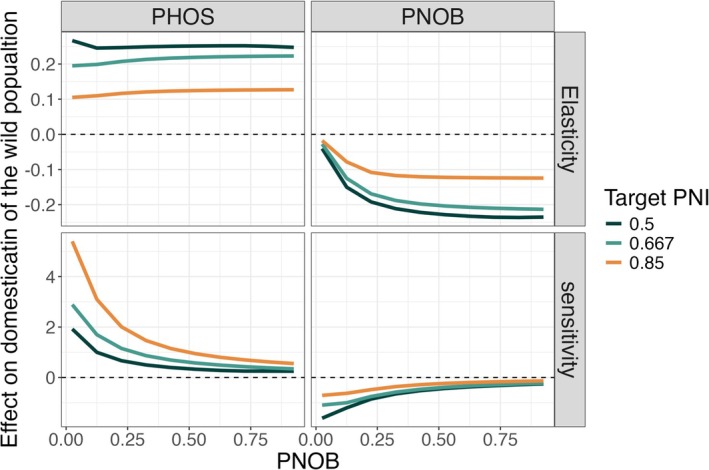
The effect of a small change in the long‐term average of pHOS and pNOB on the genotypes of the wild population, quantified by the elasticity (effect of a proportion change) and the sensitivity (effect of absolute change) of the domestication index. Please note that the elasticity and sensitivity measure derivatives of the domestication index as a function of a second variable, in this case, pHOS and pNOB. This means that the value of the sensitivities and elasticities can exceed one even though the values of the domestication index cannot.

As expected, we found that increases in pHOS increased domestication and increases in pNOB reduced domestication across scenarios (Figure 6). The scale of these effects depends on the level of integration of the hatchery program as defined by target values of pNOB, the target level of genetic impacts PNI, and whether the implementation errors were proportional changes or absolute changes (elasticity vs. sensitivity, Figure 6). The effect of a proportional change in pHOS (elasticity) was close to constant across levels of hatchery integration (pNOB), while the sensitivity to absolute changes decreased rapidly as a function of pNOB. The effect of a proportional change in pNOB increased with the level of hatchery integration, but the sensitivity (absolute change) to changes in pNOB was smaller than the sensitivity to changes in pHOS across all mitigation strategies (Figure 6). These relationships between management strategy were consistent across several alternative sets of parameters for the quantitative genetic model including higher and lower values for the strength of the fitness tradeoff $\mathrm{RR}S^{*}$ and selection strength $\sigma_{i}^{2}$ (Figure S4). We do not expect the life history parameters to influence these results because they only impact the short term, transient dynamics of the model.

### 3.3 Empirically Observed Implementation Errors

**FIGURE 7:**
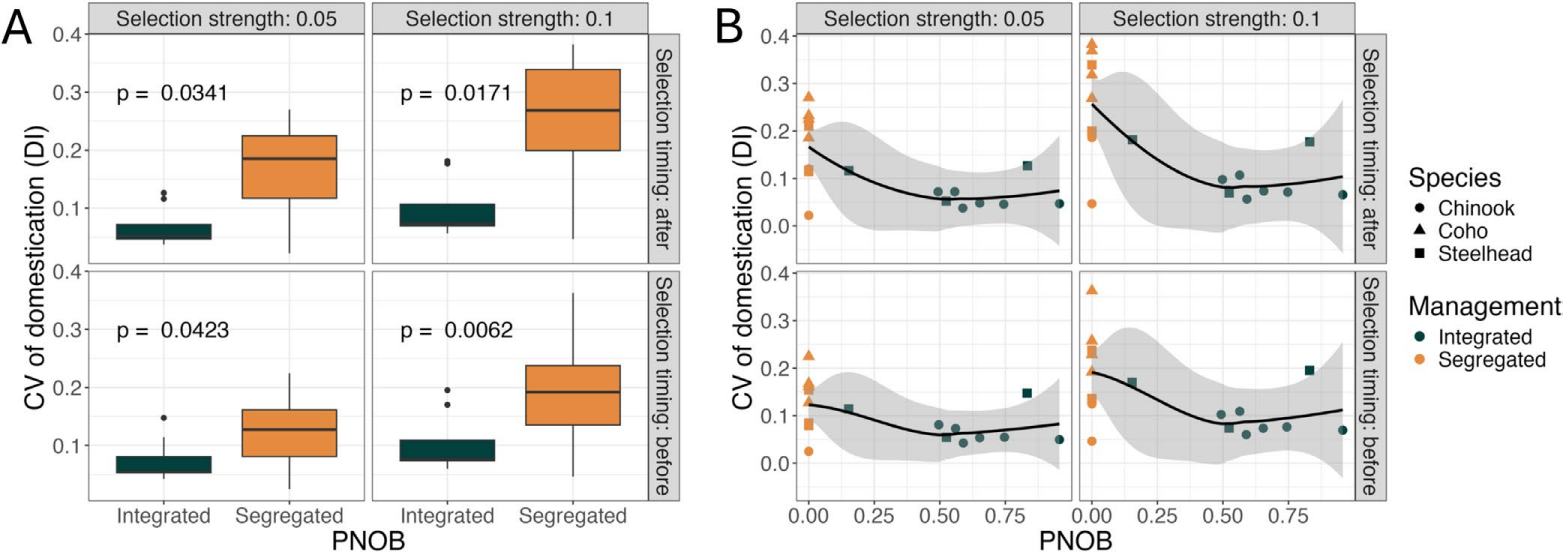
The effect of demographic variation on the variability of the domestication index as a function of the hatchery's integration level (average proportion of hatchery origin spawners). Each data point represents results from a simulation parameterized to match observed levels of demographic variation from salmon hatchery programs in Washington or Oregon. Each subpanel shows the results of a simulation with different levels of selection strength and timing in the life cycle. (Panel A) shows the difference between segregated programs (pNOB = 0) and integrated programs (pNOB > 0). The *p*‐values shown in this panel were calculated using the Kruskal‐Wallis test. (Panel B) shows the relationship between the simulated level of variation in the level of domestication and the average value of pNOB. We applied the base value of the biological parameter values listed in Table 2 for each species. We simulated demographic variability for each program using the parameter estimates listed in Tables S3 and S4.

Our theoretical analysis indicates that the ability of integrated hatchery programs to mitigate genetic impacts of the hatchery on wild populations was more robust to implementation errors than segregated hatcheries under a wide range of biological parameters and sources of implementation error. This pattern was also present under empirically observed levels of observation errors. We found that integrated programs were more robust than segregated ones on average (smaller coefficient of variation in the domestication index, Figure 7A). Still, there was significant overlap in the range of values between the two mitigation strategies. Although we found that integrated programs were more robust to the empirically observed level of demographic variability than segregated ones, differences in the value of pNOB between integrated programs had a negligible effect (Figure 7B).

One factor contributing to these observed differences is the difference in species composition. The segregated programs include three hatchery programs with coho salmon, which are not represented in the integrated programs. These populations could be biasing the estimates of variability in segregated programs upward, if implementation errors are inherently larger for coho salmon or produce large genetic changes in our model. To test the latter mechanisms, we re‐ran the simulation using the life history parameters for Chinook salmon for every hatchery program, thus controlling for the effects of species composition in the two groups. We found that controlling for life history parameters reduced but did not eliminate the difference between segregated and integrated programs (Figure S5). In addition, the estimates of pNOB in our dataset could include unmarked hatchery‐origin fish, biasing the mean value of pNOB used in our simulations upwards. We tested the impact of this hypothetical bias on our results by reducing the mean value of pNOB in our simulations for each program by 15% (detailed calculations given in Appendix S6). Accounting for this potential bias had no qualitative impact on our findings (Figure S6).

## 4 Discussion

Based on our model, we find that integrated hatchery programs are generally more robust than segregated programs to imperfect implementation of pHOS and pNOB targets. Temporary fluctuations in pHOS and pNOB cause smaller changes in the domestication of wild‐origin fish in integrated compared to segregated programs. However, in our model, these temporary fluctuations have a limited influence on the long‐term average level of domestication (Figure 4). Our findings also show that long‐term deviations from pHOS and pNOB targets can have a smaller effect in integrated than in segregated hatchery programs, but this finding was sensitive to the metric used to quantify the relationship (Figure 6, sensitivity vs. elasticity). Relatively small levels of hatchery integration were required to increase the robustness of the program to several forms of implementation error. For example, in our base parameter set, a pNOB of 25% is associated with about a 50% reduction in the sensitivity of genotypes of the wild population to short‐term variability around target values of pHOS (Figure 5 panel A). This pattern was further confirmed by simulating the model under empirically observed levels of implementation error (Figure 7).

### 4.1 Mechanistic Interpretation

The level of domestication of wild populations exposed to hatcheries is determined by the balance of gene flow between the hatchery and wild populations and selection in their respective environments. The effect of gene flow on genotypes can operate on a much faster time scale than the effects of domestication or natural selection. This explains why we find that segregated hatchery programs are a less robust strategy for mitigating domestication of the wild population. While they aim to minimize the genetic impacts of a hatchery by reducing gene flow to the wild population, the potential consequences will accumulate quickly if gene flow does in fact occur. In comparison, integrated hatchery programs reduce genetic impacts on wild populations by limiting the domestication of hatchery populations. This approach relies on controlling the balance of gene flow and local adaptation between hatchery and wild populations. Over the long term (tens of generations), a shift in the balance of gene flow and local adaptation to favor the hatchery over the wild population (an increased pHOS or decrease in pNOB) will cause both populations to become domesticated. However, in the short term, such a shift would have a limited impact on the wild population because multiple generations will be required for the hatchery population to adapt to its local environment before it can impact the wild population. This process, which requires local adaptations and several generations to accumulate, will be inherently more robust to short‐term fluctuations in the rates of gene flow between the two populations.

### 4.2 Implications for Management

Our findings can inform decisions for integrated hatchery programs seeking to minimize the demographic impacts of brood stock collection on wild populations. These findings suggest that mitigation strategies that are relatively robust to implementation errors can be achieved with a relatively small proportion of wild brood stock, provided that the proportion of hatchery‐origin spawners can also be reduced to meet the overall PNI target. However, the PNI target itself may require a higher proportion of wild broodstock. Our findings also suggest a link between the breeding strategy used by hatchery programs and the most effective approach to monitoring and evaluation. Genetic impacts on wild populations can accumulate more quickly if demographic targets (e.g., the proportion of hatchery‐origin spawners in the wild) are not met in segregated hatchery programs or in integrated programs with small average values of pNOB (< 25%). Under these conditions, frequent monitoring and evaluation of management implementation could help avert unintended genetic impacts on wild populations. In contrast, because integrated programs are more robust, monitoring and evaluation can operate on a slower time scale (i.e., less frequent adjustments of the managed strategy) for an analogous level of risk tolerance.

Robustness is one consideration involved in determining the best hatchery management approach in a given scenario, where additional considerations include the overall efficacy, feasibility, and cost of each approach. In theory, segregated hatchery programs can have very limited fitness and demographic impacts in the extreme case when they are sufficiently adapted to captivity that their reproductive success in the wild is close to zero (but this efficacy can decline precipitously with a small amount of reproductive success; Baskett and Waples 2013) or if their abundance in the wild is very well controlled. Integrated hatchery programs always have some demographic impact on wild populations because they require the capture of wild‐origin brood stock. The costs associated with integrated programs can be viewed like an insurance policy, where a small but predictable cost is paid to prevent larger, unpredictable costs. However, like insurance, the premiums are only justified if the risks are sufficiently large, which might not always be the case. Furthermore, if a hatchery population does have very low reproductive success in the wild, incorporating wild‐origin fish into the hatchery brood stock could increase genetic risks, at least temporarily, by increasing the reproductive success of hatchery‐origin fish in the wild and thus gene flow. These impacts are most likely to occur in the short term while the process of domestication is reversed and therefore do not appear in our simulations, which focus on long‐term outcomes (e.g., tens of generations).

### 4.3 Robustness and Resilience

Our model quantifies the robustness of each mitigation strategy in terms of the ecological resistance of the wild population's genetic state, where we define resistance as the amount by which an external shock changes the state of a system. Resistance represents one component of robustness, which also includes resilience, defined as the propensity of a system to return to a given state, including the potential to avoid irreversible outcomes (Levin and Lubchenco 2008). Simplifying assumptions made in our model preclude us from evaluating the potential for irreversible outcomes. For example, our model is based on quantitative genetics and therefore assumes all the relevant genetic differences between hatchery and wild populations can be explained by small changes in allele frequencies at many loci. However, some ecologically relevant traits, such as run timing, can be heavily influenced by individual loci (Narum et al. 2004; Prince et al. 2017; Thompson et al. 2019) which could go to fixation, resulting in permanent loss of genetic variation if the impacts of hatcheries are sufficiently large. Furthermore, our model only describes the population's genetics. However, genetic impacts of the hatchery can cause wild populations to decline in abundance if the fitness effects are sufficiently large. In this context, feedback between the abundance of the wild population and the genetic impact of that hatchery can reduce the likelihood of recovery (Ronce and Kirkpatrick 2001; Tufto 2001). Our findings suggest that these detrimental long‐term outcomes are less likely to occur in integrated hatchery programs because imperfect implementation of demographic targets in integrated programs causes smaller and slower changes in the genetic state of wild populations compared to segregated programs. This reduces the likelihood that the level of hatchery impacts exceeds thresholds where it becomes irreversible and allows more time for management to adapt so that undesired outcomes do not occur.

### 4.4 Model Assumptions and Limitations

Our modeling framework makes many standard assumptions about the genetic and evolutionary mechanisms that underlie the genetic impacts of hatchery and aquaculture programs on wild conspecifics (Tufto 2001; Ford 2002; Baskett and Waples 2013; Withler et al. 2018; Yang et al. 2019; Buckner et al. 2024). Like these previous studies, our study conceptualizes hatchery‐wild interactions as a system characterized by gene flow (between hatchery and wild populations) and local adaptation (to the hatchery and natural environment, respectively). This framework captures the fitness trade‐offs between hatchery and natural environments documented for many species of salmonids (Araki et al. 2007; Christie et al. 2012, 2014; Koch and Narum 2021) in a parsimonious way. However, it leaves out other mechanisms, such as the effect of hatchery production on the distribution of family sizes and thus genetic diversity of the population, and the influence of the hatchery on mate choice and mating systems. While these mechanisms are important for successful hatchery management, they are determined by the numbers and pairing of individuals used in the hatchery program (Waples et al. 2016; Oregon Hatchery Research Center Board 2024), which are beyond the scope of our study. Other factors, such as when selection occurs in the life cycle and the spawning habitats used by wild and hatchery fish, can also influence how much variation in demographic quantities (e.g., pHOS and pNOB) results in gene flow. We incorporate this into the model by exploring different timings of selection in the life cycle, which did not affect our conclusions. However, we do not include other factors like selective mating, which can reduce gene flow between populations but has a smaller effect than the timing of selection (Baskett and Waples 2013).

## 5 Conclusions

We used a quantitative population genetic model to evaluate the robustness of alternative strategies for mitigating the genetic impacts of salmon hatchery programs on wild populations to imperfect implementation. We found that under a wide range of conditions, integrated hatchery programs were more robust to imperfect implementation than segregated hatchery programs. These findings suggest that integrated hatchery programs might be a more effective strategy for mitigating the genetic impact of hatcheries in contexts where the relative abundance of wild and hatchery‐origin fish is hard to control. Furthermore, segregated hatchery programs can require more intensive adaptive management to prevent unintended outcomes.

## Conflicts of Interest

The authors declare no conflicts of interest.

## Supporting information


**Data S1:** Supporting Information.

## Data Availability

The code used to implement the quantitative genetic model and the simulation analyses can be found on github at https://github.com/Jack‐H‐Buckner/hatchery‐management‐genetic‐model.git.
