# Supplementary material for: Integrated Salmon Hatcheries Can Pose Less Genetic Risk to Wild Populations Than Segregated Programs, Given Imperfect Implementation
Source: Evol Appl. 2025 Dec 2;18(12):e70184. doi: 10.1111/eva.70184 (PMC12670298; doi:10.1111/eva.70184)
Supplement: Supplementary file 1 — Data S1: Supporting Information. [file EVA-18-e70184-s001.docx]

**Supplemental information:** Integrated salmon hatcheries can pose less genetic risk to wild populations than segregated programs, given imperfect implementation

Jack H. Buckner, Michael J. Ford, and Marissa L. Baskett

**Supplemental figures:**

| 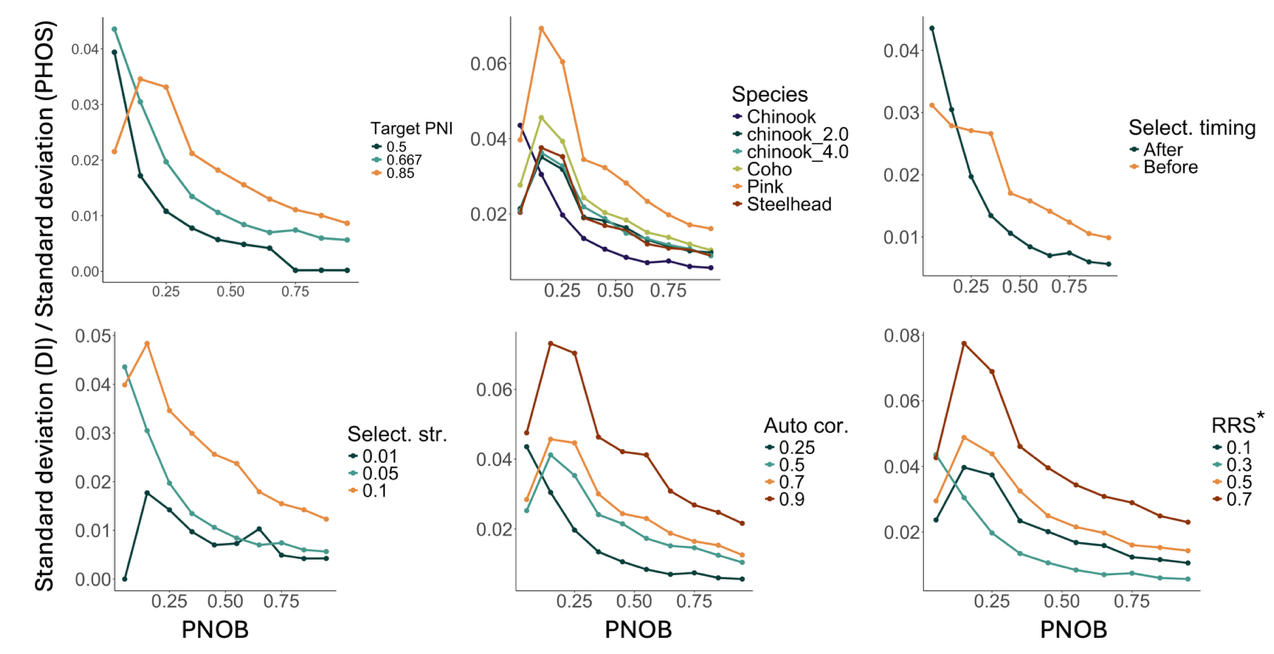 |
| --- |
| **Figure S1:** The effect of alternative model parameters on the relationship between the hatchery mitigation strategy and robustness, holding the standard deviation of demographic variability (pHOS) constant. Each panel varies a different model parameter: A) the target proportion of natural influence, B) the population’s age structure parameters, C) the timing of selection in life cycle (before or after immigration to spawning grounds), D) the strength of natural and domesticating selection, E) the autocorrelation of demographic variability, and F) the strength of fitness tradeoff between hatchery and wild). Note that selection strength is the inverse of selection variance $\frac{1}{\sigma^{2}}$. |

| **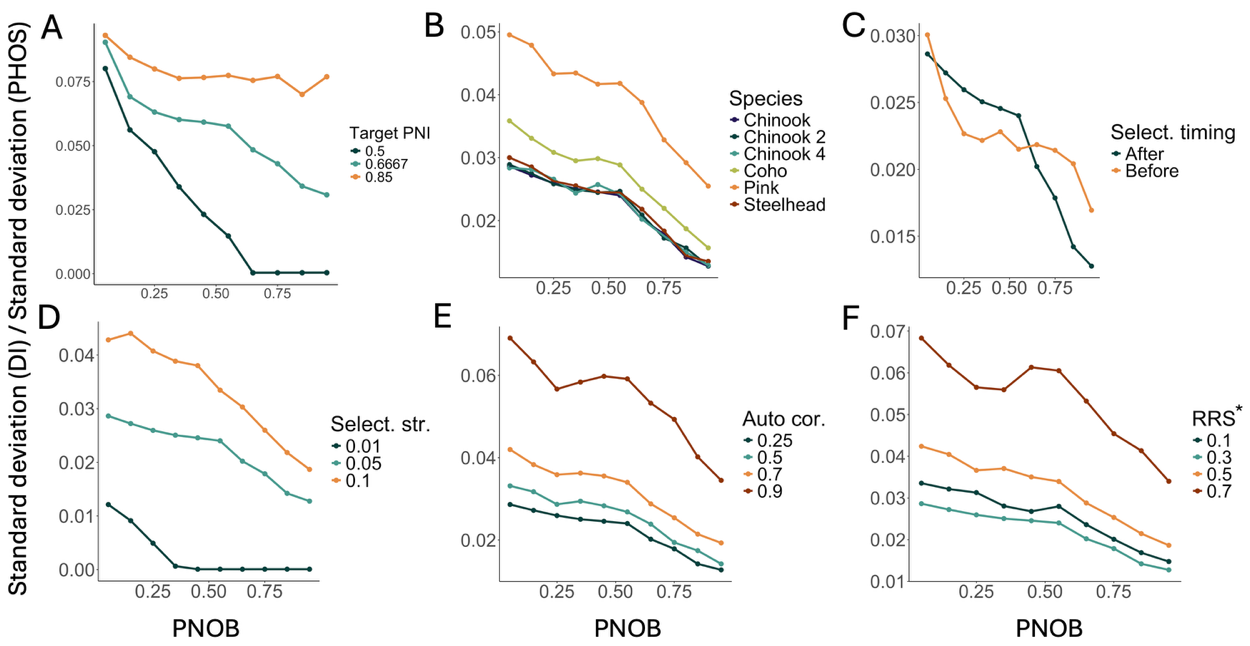** |
| --- |
| **Figure S2:** The effect of alternative model parameters on the relationship between the hatchery mitigation strategy and robustness, holding the coefficient of demographic variation (pHOS) constant. Each panel varies a different model parameter: A) the target proportion of natural influence, B) the population’s age structure parameters, C) the timing of selection in life cycle (before or after immigration to spawning grounds), D) the strength of natural and domesticating selection, E) the autocorrelation of demographic variability, and F) the strength of fitness tradeoff between hatchery and wild). Note that selection strength is the inverse of selection variance $\frac{1}{\sigma^{2}}$. |

| **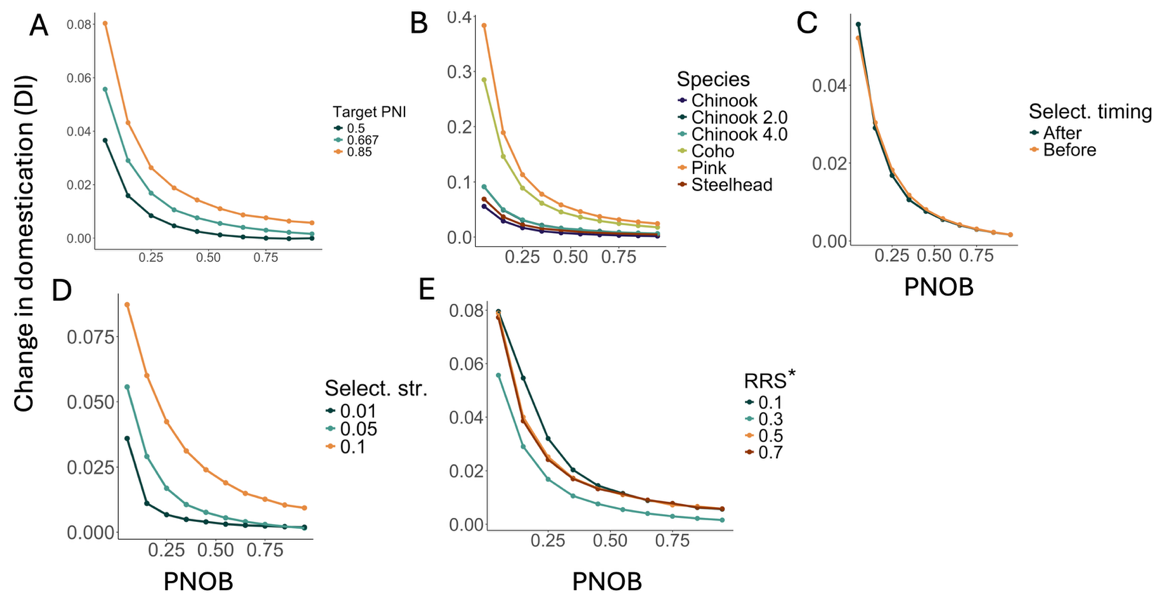** |
| --- |
| **Figure S3:** The effect of model parameters on the relationship between the hatchery management strategy and robustness to a single year with high values of pHOS. Each panel varies a different model parameter: A) the target proportion of natural influence, B) the population’s age structure parameters, C) the timing of selection in the life cycle (before or after immigration to spawning grounds), D) the strength of natural and domesticating selection, and E) the strength of the fitness tradeoff between hatchery and wild. Note that selection strength is the inverse of selection variance $\frac{1}{\sigma^{2}}$. |

| **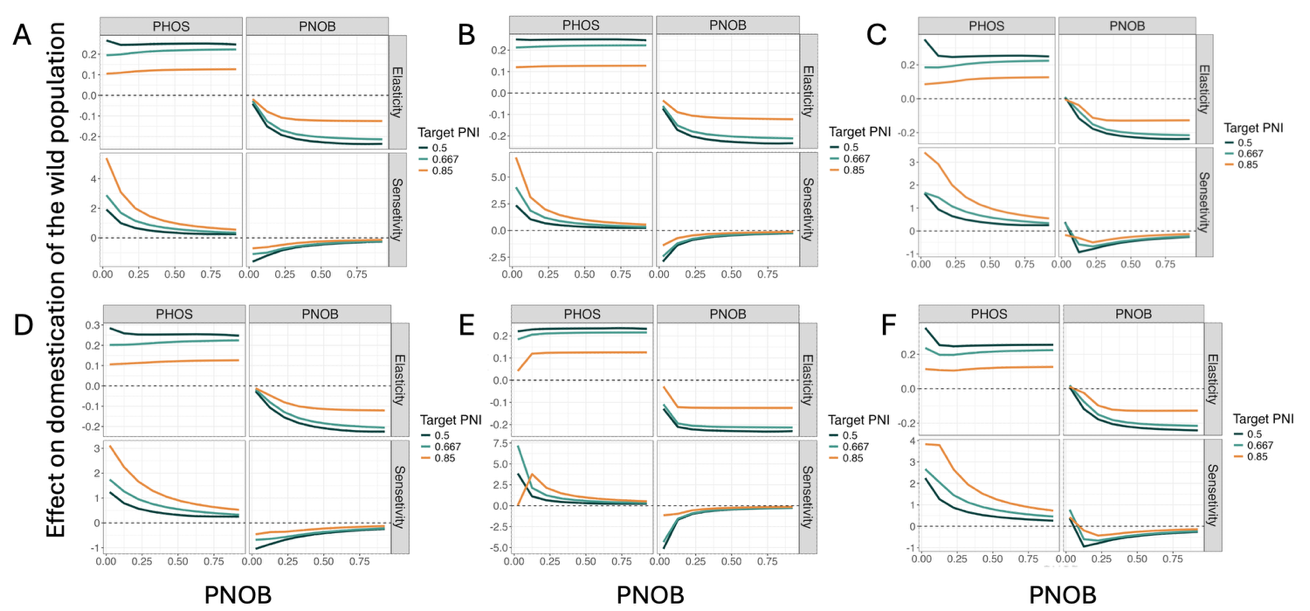** |
| --- |
| **Figure S4:** The impact of altering the model parameters on the relationship between the hatchery management strategy and its sensitivity to long-run implementation errors. Each panel shows a different parameter set: A) base parameters set, B) higher relative fitness RRS^*^ = 0.7, C) lower relative fitness RRS^*^ = 0.1, D) more selection curvature $\sigma^{2}=10,$E) less selection curvature $\sigma^{2}=100,$ F) selection acts during reproduction. Each of the four subpanels shows the effect of varying pHOS and pNOB quantified by the sensitivity and elasticity metrics. |

| **** |
| --- |
| **Figure S5:** The effect of demographic variation on the variability of the domestication of wild-origin individuals as a function of the hatchery’s integration level (average proportion of hatchery-origin spawners). Each data point represents results from a simulation parameterized to match observed levels of demographic variation from salmon hatchery programs in Washington or Oregon. Life history parameters for Chinook salmon are used in each simulation to control for differences between species. Each subpanel shows the results of a simulation with different levels of selection strength and timing in the life cycle. Panel A shows the difference between isolated programs (pNOB = 0) and integrated programs (pNOB > 0). The p-values shown in this panel were calculated using the Kruskal-Wallis test. Panel B shows the relationship between the simulated level of variation in the level of domestication and the observed average value of pNOB. |
| **** |
| **Figure S6:** The effect of demographic variation on the variability of the domestication of wild origin individuals as a function of the hatchery’s integration level (average proportion of hatchery origin spawners). Each data point represents results from a simulation parameterized to match observed levels of demographic variation from salmon hatchery programs in Washington or Oregon. Each subpanel shows the results of a simulation with different levels of selection strength and timing in the life cycle. Panel A shows the difference between isolated programs (pNOB = 0) and integrated programs (pNOB > 0). The p-values shown in this panel were calculated using the Kruskal-Wallis test. Panel B shows the relationship between the simulated level of variation in the level of domestication and the observed average value of pNOB. We multiplied the estimated values of pNOB by 0.85 for these simulations to test the effects of upward biases in the PNOB data on our findings**.** |

**SI appendix 1:** *Modeling selection on genotypes*

Selection acts on phenotypes, which we model as the sum of an individual’s genotype $g$

and environmental effects $E$

S1.1) $p= g+$ $E.$

We assume that environmental effects are normally distributed with mean zero and variance $V_{E}$. We model the probability of surviving a selection event with a quadratic fitness function with optimum trait $\theta$ and selection variance $\sigma_{p}^{2}$

S1.2) $s\left( p \right)=e^{-\frac{1}{2}\left( \frac{p-\theta}{\sigma_{p}} \right)^{2}}$.

Given the relationship between survival and phenotypes, we can calculate the probability of survival given an individual’s genotype by integrating over the distribution of environmental effects

S1.3) $s\left( g \right)=\int_{-\infty}^{\infty} s(g+E)\frac{1}{\sqrt{2\pi V_{E}}}e^{-E^{2}/(2V_{E})}dE$,

We solve by substituting equation S2 into equation S3 and simplifying yields

S1.4) $s\left( g \right)=\frac{1}{\sqrt{2\pi V_{E}}}\int_{-\infty}^{\infty} e^{-\frac{1}{2}\left( \frac{g+E-\theta_{i}}{\sigma_{p,i}} \right)^{2}}e^{-E^{2}/(2V_{E})}dE$

S1.5) $s\left( g \right)=\frac{1}{\sqrt{2\pi V_{E}}}\int_{-\infty}^{\infty} e^{-\frac{1}{2}\left( \left( \frac{g+E-\theta_{i}}{\sigma_{p,i}} \right)^{2}+\frac{E^{2}}{V_{E}} \right)}dE$.

We solve the integral in equation S1.5 by re-writing the quadratic function in the exponent in the form of a normal distribution plus a constant

S1.6) $\left( \frac{g+E-\theta_{i}}{\sigma_{p,i}} \right)^{2}+\frac{E^{2}}{V_{E}}=\frac{\left( E-\mu\right)^{2}}{\sigma_{i}^{2}}+c$

First, we expand both sides of equation S1.6

S1.7) $E^{2}\left( \frac{1}{V_{E}}+\frac{1}{\sigma_{p,i}^{2}} \right)+2E\frac{\left( g-\theta_{i} \right)}{\sigma_{p,i}^{2}}+\frac{\left( g-\theta_{i} \right)^{2}}{\sigma_{p,i}^{2}}=\frac{E^{2}}{\sigma_{i}^{2}}-\frac{E\mu}{\sigma_{i}^{2}}+\frac{\mu^{2}}{\sigma_{i}^{2}}+c$

Next we can solve for $\sigma^{2}$ using the $E^{2}$ term

S1.8) $\frac{E^{2}}{\sigma_{i}^{2}}=\left( \frac{1}{V_{E}}+\frac{1}{\sigma_{p_{i}}^{2}} \right)E^{2} \to{\sigma\_i}^{2}={\left( \frac{1}{\sigma_{p,i}^{2}}+\frac{1}{V_{E}} \right)^{-1}}$

Next we solve for $\mu$ using the $\mu E$ term and the value of $\sigma^{2}$

S1.9) $\frac{E\mu}{\sigma_{i}^{2}}= -\frac{1}{\sigma_{p,i}^{2}}E\left( g-\theta_{i} \right) \to\mu= -\frac{\frac{1}{\sigma_{p,i}^{2}}\left( g-\theta_{i} \right)}{\left( \frac{1}{\sigma_{p,i}^{2}}+\frac{1}{V_{E}} \right)}$

Finally, we solve for $c$ using the $\mu^{2}$ term

S1.10) $\frac{\mu^{2}}{\sigma_{i}^{2}}+c= \frac{\frac{1}{\sigma_{p,i}^{2}}\left( g-\theta_{i} \right)^{2}}{\left( \frac{1}{\sigma_{p,i}^{2}}+\frac{1}{V_{E}} \right)^{2}} \left( \frac{1}{\sigma_{p,i}^{2}}+\frac{1}{V_{E}} \right)+c=\frac{\left( g-\theta_{i} \right)^{2}}{\sigma_{p,i}^{2}\left( 1+\sigma_{p,i}^{2}/V_{E} \right)} +c=\frac{\left( g-\theta_{i} \right)^{2}}{\sigma_{p,i}^{2}}$

S1.11) $c=\frac{\left( g-\theta_{i} \right)^{2}}{\sigma_{p,i}^{2}\left( 1+\sigma_{p,i}^{2}/V_{E} \right)}-\frac{\left( 1+\sigma_{p,i}^{2}/V_{E} \right)\left( g-\theta_{i} \right)^{2}}{\sigma_{p,i}^{2}\left( 1+\sigma_{p,i}^{2}/V_{E} \right)}$

S1.12) $c=\frac{\sigma_{p,i}^{2}/V_{E}\left( g-\theta_{i} \right)^{2}}{\sigma_{p,i}^{2}\left( 1+\sigma_{p,i}^{2}/V_{E} \right)}$

S1.13) $c=\frac{\left( g-\theta_{i} \right)^{2}}{\left( V_{E}+\sigma_{p,i}^{2} \right)}$

This allows us to rewrite equation S1.5 as the integral of a normal density function over the environmental effects $E$ times a constant that is a function of genotypes

S1.14) $s\left( g \right)=\frac{1}{\sqrt{2\pi V_{E}}}\int_{-\infty}^{\infty} e^{-\frac{1}{2}\left( \left( \frac{E-\mu}{\sigma_{i}} \right)^{2}+\frac{\left( g-\theta_{i} \right)^{2}}{\left( V_{E}+\sigma_{p,i}^{2} \right)} \right)}dE$.

S1.15) $s\left( g \right)=\frac{1}{\sqrt{2\pi V_{E}}}e^{-\frac{1}{2}\frac{\left( g-\theta_{i} \right)^{2}}{\left( V_{E}+\sigma_{p,i}^{2} \right)}}\int_{-\infty}^{\infty} e^{-\frac{1}{2}\left( \frac{E-\mu}{\sigma_{i}} \right)^{2}}dE$.

We can solve the integral over $E$ using the normalizing constant from a normal distribution with variance $\sigma_{i}^{2}$ yielding a closed form solution for the probability of survival given an individual genotype

S1.16) $s\left( g \right)=\frac{1}{2\pi\sigma_{i}\sqrt{V_{E}}} e^{-\frac{1}{2}\frac{\left( g-\theta_{i} \right)^{2}}{\left( V_{E}+\sigma_{p,i}^{2} \right)}}$

Re-naming the parameters yields main text equation 5

S1.17) $s\left( g \right)={s_{max}e}^{-\frac{1}{2}\left( \frac{g-\theta_{i}}{\sigma_{i}} \right)^{2}}$.

Where $s_{max}$ is the probability of survival given the optional phenotype $\theta_{i}$ and $\sigma_{i}$ is the effective selection strength on genotypes accounting for imperfect heritability of the trait.

S1.18) $s_{max}=\frac{1}{2\pi\sigma_{i}\sqrt{V_{E}}}$

S1.19) $\sigma_{i}=\left( V_{E}+\sigma_{p,i}^{2} \right)^{1/2}$*.*

**Supplemental appendix 2:**

The distribution of genotypes after selection is given the normalized product of the initial genotype distribution $\psi(g)$ times the selection function $s\left( g \right)$. Given the findings of Turelli and Barton (1994) we have chosen to approximate the genotype distribution $\psi(g)$ with a normal probability density function with mean $\bar{g}$ and variance $V$

S2.1) $\psi\left( g \right)=\frac{1}{\sqrt{2\pi V}}e^{-\frac{\left( g-\bar{g} \right)^{2}}{2V}}$.

The genotype distribution after selection $\psi'\left( g \right)$is the product of equation S2.1 and S1.17 times a normalization constant $Z$

S2.2) $\psi^{'}(g)=\frac{{\psi\left( g \right)}s\left( g \right)}{\int{\psi\left( g \right)}s\left( g \right)dg}=Z {\frac{1}{\sqrt{2\pi V}}e}^{-\frac{\left( g-\bar{g} \right)^{2}}{2V}}s_{max}e^{-\frac{1}{2}\left( \frac{g-\theta}{\sigma} \right)^{2}}$

Because we are approximating the genotype distribution with a normal probability density, we only need to track the changes in the mean and the variance of the of the distribution. Equation S2.2 is common in Bayesian statistics, where the two function $\psi\left( g \right)$and $s\left( g \right)$ can be thought of as the prior and the likelihood functions and $\psi^{'}(g$) the posterior distribution. Because these equations have the same structure we can borrow formulas from Bayesian statistics to calculate the mean $\bar{g}^{'}$ and variance $V'$ of the updated distribution. Specifically, the “prior” distribution $\psi\left( g \right)$ is a normal distribution with mean $\bar{g}$ and variance $V$ and the likelihood is normal with mean $\theta$ and variance $\sigma^{2}$. Appling the formulas for a normal prior and likelihood yields main text equations 5 and 6 reproduced below in simplified notation

S2.4) $\bar{g}^{'}=\left( \frac{1}{V}+\frac{1}{\sigma^{2}} \right)^{-1}\left( \frac{\bar{g}}{V}+\frac{\theta}{\sigma^{2}} \right),$

S2.5) $V_{0,i,t}^{'}=\left( \frac{1}{V}+\frac{1}{\sigma^{2}} \right)^{-1}.$

**Supplemental appendix 3:** Abundance and fecundity at age parameters

We queried the RMIS database (Johnson 2004)to obtain samples of the age and length of hatchery-origin fish observed in spawning ground surveys (RMIS fishery codes 50 -59) between the years 2014 and 2016 in the Columbia River basin (RMIS region codes “LOCR”, UPCR”, CECR”, and “SNAK”). We matched coded wire tags detected in spawning surveys to hatchery release groups, also recorded in the RMIS database, to determine the age, species, and run type of each individual in the sample.  For Chinook salmon model parameters, we calculated the proportion of fall run Chinook (species code 1, run timing code 3) in each age class. For coho salmon, we restricted our analysis to late fall run individuals (species code 2, run code 7). We used summer run fish to parametrize the life history parameters for steelhead (species code 3, run code 2).

To calculate fecundity at age, we first estimated the fecundity of each sampled individual using the fecundity at length (*L*) relationship:

$F\left( L \right)=\alpha L^{b}$

We then grouped the samples by age class and calculated the average estimated fecundity. We restricted this analysis to the same species and run timing combination used to calculate the relative abundance of each age class.

**Supplemental appendix 4:** Model parametrization from empirical estimates of implementation error

Tables S1 and S2 describe the combinations of pHOS and pNOB data used to parameterize our simulations with empirically observed levels of implementation error. We matched pNOB data to the closest available pHOS data set to construct each simulation. However, in some cases, pHOS was reported at a larger spatial scale than the pNOB data, as detailed in Table 1. Tables S3 and S4 give the precise values of the parameters used in our simulations.

**Table S1:** Sources of PHOS and PNOB data used to parametrize the demographic variability of the stochastic population genetic model for the integrated program cases.

| **Species / Run** | **PHOS data ^1^** | **PNOB data** | **Facility** |
| --- | --- | --- | --- |
| Summer Steelhead | Wenatchee R. Steelhead | Wenatchee R. | Eastbank^2^ |
|  | Methow R. Steelhead | Methow River | Wells^2^ |
|  |  | Twisp | Methow^2^ |
|  | Deschutes River | N.A. | Round Butte^3^ |
|  | N.A. | Tucannon River | Lyons Ferry^2^ |
| Spring Chinook | Wenatchee R. Chinook^2^ | Nason | Eastbank^2^ |
|  |  | Chiwawa | Eastbank^2^ |
|  | Methow R. Chinook | Twisp River | Methow^2^ |
|  |  | Methow River | Methow^2^ |
|  | Tucannon R. Chinook | Tucannon River | Lyons Ferry^2^ |
|  | Cascade River | N.A. | Marblemount Hatchery^2^ |
| Summer Chinook | Wenatchee R. Chinook^2^ | Wenatchee | Eastbank^2^ |

^1.^ PHOS estimates likely include fish from hatchery programs not included in the PNOB data. ^2.^ Program management based on WDFW hatchery reform report (Appendix B of Murdoch 2020).

**Table S2:** sources of PHOS data used to parameterize the model for the isolated program cases.

| **Species / Run** | **PHOS data ^1^** | **Facility** |
| --- | --- | --- |
| Fall Chinook Salmon | Nooksack River | Glenwood Springs^1^ |
|  |  | Samish^1^ |
| Winter Steelhead | Clackamas River | Clackamas2 |
| Summer Steelhead | Deschutes River | Round Butte^3^ |
| Coho | North Umpqua River | Rock Creek^4^ |
|  | South Umpqua River | Rock Creek^4^ |
|  | Nehalem River | Nehalem^5^ |
|  | Tillamook River | Trask^6^ |

^1.^ Isolated management based on WDFW hatchery reform report (Appendix B of Murdoch 2020)^2.^ Clackamas Winter steelhead HGMP from ODFW website. ^3.^ Deschutes Summer steelhead HGMP from ODFW website ^4.^ Rock Creek / Umpqua Basin Coho HGMP from ODFW website ^5.^ Nehalem River Coho HGMP from ODFW website ^6.^ Trask Coho HGMP from ODFW website

**Table S3:** pHOS variability parameters

| **Species / Run** | **pHOS data ^1^** | **Mean** $\mu$ | **Variance** $\boldsymbol{\tau}$ | **Autocorrelation** $\rho$ |
| --- | --- | --- | --- | --- |
| Summer Steelhead | Wenatchee R. Steelhead | -0.30 | 1.01 | 0.61 |
|  | Methow R. Steelhead | -1.39 | 1.10 | 0.80 |
|  | E. Deschutes River | -1.42 | 1.25 | 0.61 |
|  | W. Deschutes River | -1.87 | 1.45 | 0.82 |
| Spring Chinook | Wenatchee R. Chinook^2^ | -0.34 | 1.05 | 0.40 |
|  | Methow R. Chinook | -0.22 | 1.16 | 0.74 |
|  | Tucannon R. Chinook | 0.00 | 1.14 | 0.29 |
| Fall Chinook | Nooksack River | 1.26 | 1.41 | 0.80 |
|  | Cascade River | -2.62 | 2.00 | -0.25 |
| Winter Steelhead | Clackamas River | -1.61 | 1.23 | 0.54 |
| Coho | North Umpqua River | -1.47 | 3.57 | 0.93 |
|  | South Umpqua River | -2.64 | 1.64 | 0.25 |
|  | Nehalem River | -2.87 | 2.19 | 0.54 |
|  | Tillamook River | -2.70 | 1.57 | 0.54 |

**Table S4:** pNOB variability parameters

| **Species / Run** | **pNOB data** | **Facility** | **Mean** $\mu$ | **Variance** $\boldsymbol{\tau}$ | **Autocorrelation** $\rho$ |
| --- | --- | --- | --- | --- | --- |
| Summer Steelhead | Wenatchee R. | Eastbank^2^ | 0.10 | 0.33 | 0.26 |
|  | Methow River | Wells^2^ | -2.77 | 2.06 | 0.34 |
|  | Twisp | Methow^2^ | 2.13 | 1.40 | 0.13 |
|  | Tucannon River | Lyons Ferry^2^ | 1.15 | 1.61 | 0.31 |
| Spring Chinook | Nason | Eastbank^2^ | 1.71 | 1.93 | 0.32 |
|  | Chiwawa | Eastbank^2^ | 1.02 | 1.92 | 0.22 |
|  | Twisp River | Methow^2^ | -0.03 | 1.49 | 0.26 |
|  | Methow River | Methow^2^ | 0.45 | 2.28 | 0.16 |
|  | Tucannon River | Lyons Ferry^2^ | 0.44 | 1.02 | 0.32 |
| Summer Chinook | Wenatchee | Eastbank^2^ | 5.30 | 2.52 | 0.15 |

**Supplemental appendix 5:** Hierarchical model for pHOS and pNOB time series

We used hierarchical Bayesian autoregressive state space models to estimate the mean, variance, trend, and autoregression parameters for the pHOS and pNOB time series. We used separate models for the pHOS and pNOB data because these data were reported in different ways. The pHOS data were reported in terms of the estimated proportion of hatchery-origin fish based on surveys of salmon spawning grounds. The pNOB data were reported in terms of the raw number of individuals of hatchery and wild-origin used in the program's brood stock. We constructed a model with a different observation process to accommodate these differences in the data.

pHOS model

We applied a log-odds (logit) transform to the pHOS time series mapping the data from the interval $\left( 0,1 \right)$to the full real line $\left( -\infty,\infty\right)$. This transformation made the data roughly normally distributed allowing us to use standard autoregressive models. We modeled the transformed data $x_{i,t}^{H}$ for each population $i$ and year $t$ as the sum of a population level mean $\mu_{i}^{H}$, a linear trend $\beta_{i}^{H}\left( t-\bar{t}_{i} \right)$ and year effects $\epsilon_{i,t}^{H}$ that follow and AR1 distribution with correlation coefficient $\rho_{i}^{H}$ and variance $\tau_{i}^{H}$:

$$x_{i,t}^{H}=\log\left( \frac{PHOS_{i,t}}{1-PHOS_{i,t}} \right) .$$

$$x_{i,t}^{H}=\mu_{i}^{H}+ \beta_{i}^{H}\left( t-\bar{t}_{i} \right)+\epsilon_{i,t}^{H}$$

$$\epsilon_{i,t}^{H}=\rho_{i}^{H}\epsilon_{i,t-1}^{H}+\nu_{i,t}^{H}$$

$$\nu_{i,t}^{H}\sim N(0,\tau_{i}^{H})$$

pNOB model

We used a different approach to model the pNOB data because many years contained observations equal to either zero or one. This prevented the use of the log odds transform which is undefined for these values. Instead, we modeled the total number of wild-origin brood stock $NOB_{i,t}$ with a binomial distribution:

$$NOB_{i,t}\sim\mathrm{Binom}\left( p_{i,t},NOB_{i,t}+HOB_{i,t} \right)$$

where $p_{i,t}$ is the probability a fish in the brood stock of program $i$ in year $t$ is wild-origin and $HOB_{i,t}$ is the number of hatchery-origin brood stock. We modeled variation in the log-odds transformed probability parameters $x_{i,t}^{N}$as the sum of the program mean $\mu_{i}^{N}$, a linear trend $\beta_{i}^{N}\left( t-\bar{t}_{i} \right)$ and autocorrelated year effects $\epsilon_{i,t}^{N}$. Combining these assumptions yields a system of four equations:

$$p_{i,t}= \mathrm{logi}t^{-1}\left( x_{i,t}^{N} \right)$$

$$x_{i,t}^{N}=\mu_{i}^{N}+ \beta_{i}^{N}\left( t-\bar{t}_{i} \right)+\epsilon_{i,t}^{N}$$

$$\epsilon_{i,t}^{N}=\rho_{i}^{N}\epsilon_{i,t-1}^{N}+\nu_{i,t}^{N}$$

$$\nu_{i,t}^{N}\sim N\left( 0,\tau_{i}^{N} \right),$$

where $\rho_{i}$ is the autocorrelation coefficient and $\tau_{i}$ is the variance parameter of an AR1 process.

Hierarchical priors

We used hierarchical priors for each of the four parameters in the two time series models $\mu_{i}^{*},\beta_{i}^{*},\rho_{i}^{*}$ and $\tau_{i}^{*}$ ($* \in\left\{ N,H \right\}$). We used normal prior for the mean $\mu_{i}^{*}$, slope $\beta_{i}^{*}$, and autocorrelation parameters $\rho_{i}^{*}$ and a gamma distribution for the variance parameters $\tau_{i}^{*}$. For example, the autocorrelation coefficient in the pHOS model follow a normal prior with mean $\hat{\rho}^{H}$ and variance $\sigma_{\rho}^{H}$:

$$\rho_{i}^{H}\sim\mathrm{Normal}\left( \hat{\rho}^{H} , \sigma_{\rho^{H}} \right).$$

The mean and variance parameter are estimated by the model and have their own prior distributions. We use a normal prior for the mean parameter and a gamma distribution for the variance:

$$\hat{\rho}^{H}\sim\mathrm{Normal}\left( 0,\sigma_{\hat{p}^{H}} \right)$$

$\sigma_{\hat{p}^{H}}\sim\mathrm{Gamma}\left( r_{\hat{p}^{H}},\theta_{\hat{p}^{H}} \right)$,

Where $\sigma_{\hat{p}^{H}}$is the prior variance for the mean parameter and $r_{\hat{p}^{H}},\theta_{\hat{p}^{H}}$ are the rate and shape parameters for the gamma distribution. The priors for the mean and slope parameters $(\mu_{i}^{H}, \beta_{i}^{H}, \mu_{i}^{N}, \beta_{i}^{N}$) have with same structure. The priors for the variance parameters $\tau_{i}^{*}$, however, follow a gamma distribution:

$\tau_{i}^{*}\sim\mathrm{Gamma}\left( \frac{\hat{\tau}^{*}}{\sigma_{\tau^{*}}^{2}},\frac{\hat{\tau}^{*}}{\sigma_{\tau^{*}}} \right)$,

where $\hat{\tau}^{*}$ is the mean and $\sigma_{\tau^{*}}^{2}$ is the variance of the gamma distribution. These parameters in turn have gamma distribution priors respectively:

$$\hat{\tau}^{*}\sim\mathrm{Gamma}\left( r_{\hat{\tau}^{*}},\theta_{\hat{\tau}^{*}} \right)$$

$\sigma_{\hat{\tau}^{*}}\sim\mathrm{Gamma}\left( r_{\sigma_{\hat{\tau}^{*}}},\theta_{\sigma_{\hat{\tau}^{*}}} \right)$.

We used informative priors for the group mean and variance parameters (e.g. $\hat{\rho}^{H}$ and $\sigma_{\hat{p}^{H}}$ ) to facilitate posterior sampling (Table S1). We chose priors for the group mean parameters centered at zero which tends to reduce the parameter estimates. This choice in prior is called a regularizing prior and is a conservative choice in the sense that it increased the information required to detect features such as trends and autocorrelation in the data. We chose the priors for the group variance parameters (e.g. $\sigma_{p^{H}}$) with higher densities at smaller parameter values. This choice implies that we expect the characteristics of the time series to be somewhat similar a priori, which is reasonable given that the data come from populations in a single region experiencing similar abiotic and management conditions.

Model fitting

We fit the time series models to the pHOS and pNOB data separately, sampling from the posterior distribution using the No U-Turns sampler implemented in the Stan model building package (Stan Development Team 2020). We ran the sampling algorithm for four chains of length 8000, with 4000 warm-up steps and 4000 sampling steps. We used the shinystan R package to visually inspect the MCMC chains for mixing and to estimate the effective sample size. We adjusted the number of sampling iterations of the No U-Turn sampler \until the posterior samples for each model parameter had an effective sample size of at least 800.

**Table S5:** Bayesian priors

| Parameter | description | value |
| --- | --- | --- |
| $\boldsymbol{\sigma}_{{\hat{\boldsymbol{p}}}^{\boldsymbol{H}}}^{\boldsymbol{2}}$ | Prior variance of the average autocorrelation coefficient $\hat{\rho}^{H}$for the PHOS time series. | 0.01 |
| $\boldsymbol{r}_{\boldsymbol{\rho}^{\boldsymbol{H}}}$ | Rate parameter of the gamma distribution prior for the within group variance of the auto correlation coefficients $\sigma_{\rho^{H}}$ | 1.0 |
| $\boldsymbol{\theta}_{\boldsymbol{\rho}^{\boldsymbol{H}}}$ | Shape parameter of the gamma distribution prior for the within group variance of the auto correlation coefficients $\sigma_{\rho^{H}}$ | 5.0 |
| $\boldsymbol{\sigma}_{{\hat{\boldsymbol{\beta}}}^{\boldsymbol{H}}}^{\boldsymbol{2}}$ | Prior variance of the average trend parameter $\hat{\beta}^{H}$for the PHOS time series. | 0.25 |
| $\boldsymbol{r}_{\boldsymbol{\beta}^{\boldsymbol{H}}}$ | Rate parameter of the gamma distribution prior for the within group variance of the trend parameters $\sigma_{\beta^{H}}$ | 1.0 |
| $\boldsymbol{\theta}_{\boldsymbol{\beta}^{\boldsymbol{H}}}$ | Shape parameter of the gamma distribution prior for the within group variance of the trend parameters $\sigma_{\beta^{H}}$ | 5.0 |
| $\boldsymbol{\sigma}_{{\hat{\boldsymbol{\mu}}}^{\boldsymbol{H}}}^{\boldsymbol{2}}$ | Prior variance of the average mean value $\hat{\mu}^{H}$for the PHOS time series. | 1.0 |
| $\boldsymbol{r}_{\boldsymbol{\mu}^{\boldsymbol{H}}}$ | Rate parameter of the gamma distribution prior for the within group variance of the mean parameters $\sigma_{\mu^{H}}$ | 1.0 |
| $\boldsymbol{\theta}_{\boldsymbol{\mu}^{\boldsymbol{H}}}$ | Shape parameter of the gamma distribution prior for the within group variance of the mean parameters $\sigma_{\mu^{H}}$ | 5.0 |
| $\boldsymbol{r}_{{\hat{\boldsymbol{\tau}}}^{\boldsymbol{H}}}$ | Rate parameter of the gamma prior for the average variance parameter $\hat{\tau}^{H}$ of the PHOs data | 1.0 |
| $\boldsymbol{\theta}_{{\hat{\boldsymbol{\tau}}}^{\boldsymbol{H}}}$ | Shape parameter of the gamma prior for the average variance parameter $\hat{\tau}^{H}$ of the PHOs data | 1.0 |
| $\boldsymbol{r}_{\boldsymbol{\sigma}_{{\hat{\boldsymbol{\tau}}}^{\boldsymbol{H}}}}$ | Rate parameter of the gamma distribution prior for the within group variance of the PHOS variance parameters $\sigma_{\tau^{H}}^{2}$ | 1.0 |
| $\boldsymbol{\theta}_{\boldsymbol{\sigma}_{{\hat{\boldsymbol{\tau}}}^{\boldsymbol{H}}}}$ | Shape parameter of the gamma distribution prior for the within group variance of the PHOS variance parameters $\sigma_{\tau^{H}}^{2}$ | 1.0 |
| $\boldsymbol{\sigma}_{{\hat{\boldsymbol{p}}}^{\boldsymbol{N}}}^{\boldsymbol{2}}$ | Prior variance of the average autocorrelation coefficient $\hat{\rho}^{N}$for the PNOB time series. | 0.01 |
| $\boldsymbol{r}_{\boldsymbol{\rho}^{\boldsymbol{N}}}$ | Rate parameter of the gamma distribution prior for the within group variance of the auto correlation coefficients $\sigma_{\rho^{N}}$ | 1.0 |
| $\boldsymbol{\theta}_{\boldsymbol{\rho}^{\boldsymbol{N}}}$ | Shape parameter of the gamma distribution prior for the within group variance of the PNOB auto correlation coefficients $\sigma_{\rho^{N}}$ | 5.0 |
| $\boldsymbol{\sigma}_{{\hat{\boldsymbol{\beta}}}^{\boldsymbol{N}}}^{\boldsymbol{2}}$ | Prior variance of the average trend parameter $\hat{\beta}^{N}$for the PNOB time series. | 0.25 |
| $\boldsymbol{r}_{\boldsymbol{\beta}^{\boldsymbol{N}}}$ | Rate parameter of the gamma distribution prior for the within group variance of the trend parameters $\sigma_{\beta^{H}}$ | 1.0 |
| $\boldsymbol{\theta}_{\boldsymbol{\beta}^{\boldsymbol{N}}}$ | Shape parameter of the gamma distribution prior for the within group variance of the trend parameters $\sigma_{\beta^{H}}$ | 5.0 |
| $\boldsymbol{\sigma}_{{\hat{\boldsymbol{\mu}}}^{\boldsymbol{N}}}^{\boldsymbol{2}}$ | Prior variance of the average mean value $\hat{\mu}^{H}$for the PNOB time series. | 1.0 |
| $\boldsymbol{r}_{\boldsymbol{\mu}^{\boldsymbol{N}}}$ | Rate parameter of the gamma distribution prior for the within group variance of the mean PNOB parameters $\sigma_{\mu^{H}}$ | 1.0 |
| $\boldsymbol{\theta}_{\boldsymbol{\mu}^{\boldsymbol{N}}}$ | Shape parameter of the gamma distribution prior for the within group variance of the mean PNOB parameters $\sigma_{\mu^{H}}$ | 5.0 |
| $\boldsymbol{r}_{{\hat{\boldsymbol{\tau}}}^{\boldsymbol{N}}}$ | Rate parameter of the gamma prior for the average variance parameter $\hat{\tau}^{H}$ of the PNOB data | 1.0 |
| $\boldsymbol{\theta}_{{\hat{\boldsymbol{\tau}}}^{\boldsymbol{N}}}$ | Shape parameter of the gamma prior for the average variance parameter $\hat{\tau}^{H}$ of the PNOB data | 1.0 |
| $\boldsymbol{r}_{\boldsymbol{\sigma}_{{\hat{\boldsymbol{\tau}}}^{\boldsymbol{N}}}}$ | Rate parameter of the gamma distribution prior for the within group variance of the PNOB variance parameters $\sigma_{\tau^{H}}^{2}$ | 1.0 |
| $\boldsymbol{\theta}_{\boldsymbol{\sigma}_{{\hat{\boldsymbol{\tau}}}^{\boldsymbol{N}}}}$ | Shape parameter of the gamma distribution prior for the within group variance of the PNOB variance parameters $\sigma_{\tau^{H}}^{2}$ | 1.0 |

**Table S6:** No U-Turn sampler parameters

| parameter | PHOS data | PNOB data |
| --- | --- | --- |
| Chains | 4 | 4 |
| iterations | 4000 | 4000 |
| Maximum tree depth | 12 | 12 |
| Adaptive delta | 0.95 | 0.95 |

**Supplemental appendix 6:** Testing effects of bias in the pNOB data

It is possible that the estimates of pNOB from the hatchery programs we analyzed are systematically biased upward because unmarked hatchery-origin individuals were counted as wild-origin fish. These biases could only be corrected by using genetic methods that identify parentage, which we do not have access to and simply may not exist for all hatchery programs and years. To test the potential impact of these biases, we re-ran the simulation tests, reducing the estimated values of pNOB by a factor of 0.15. However, our estimates of the mean and variability of pNOB are calculated under a log odds transform. To reduce the mean value of pNOB we first transformed the mean parameters $\mu_{i}^{N}$ onto a probability scale with the inverse log-odds function, $\hat{p}_{i}=\mathrm{logit}^{-1}\left( \mu_{i}^{N} \right)$. We then multiplied the probability scale value $\hat{p}_{i}$ by the bias correction factor (1-0.15), and back transformed the result to get the bias-corrected mean parameter under the log-odds transform $\mu_{i,c}^{N}=\mathrm{logit}\left( \left( 1-0.15 \right)\hat{p}_{i} \right)$. We did not change the estimated values of the autocorrelation coefficient $\rho_{i}^{N}$or variance $\tau_{i}^{N}.$ For values of $\hat{p}_{i}>0.5$ this procedure will tend to increase the variability of pNOB in the simulations and for values of $\hat{p}_{i}<0.5$ it will tend to reduce the variability, because the log odd transform tends to squish the variance of the time series when its values are near $0$ or $1.$ The results of this analysis are shown in figure S6.

**Works cited:**

Johnson, J. K. 2004. Regional Overview of Coded Wire Tagging of Anadromous Salmon and Steelhead in Northwest America:40.

Stan Development Team. 2020. Rstan: the R interface to stan.

Turelli, M., and N. H. Barton. 1994. Genetic and statistical analyses of strong selection on polygenic traits: what, me normal? Genetics 138:913–941.
